# Effectiveness of interventions to increase healthcare workers’ adherence to vaccination against vaccine-preventable diseases: a systematic review and meta-analysis, 1993 to 2022

**DOI:** 10.2807/1560-7917.ES.2024.29.9.2300276

**Published:** 2024-02-29

**Authors:** Marco Clari, Beatrice Albanesi, Rosanna Irene Comoretto, Alessio Conti, Erika Renzi, Michela Luciani, Davide Ausili, Azzurra Massimi, Valerio Dimonte

**Affiliations:** 1 Department of Sciences of Public Health and Paediatrics, University of Turin, Turin, Italy; 2 Department of Public Health and Infectious Diseases, Sapienza University of Rome, Rome, Italy; 3 Department of Medicine and Surgery, University of Milano - Bicocca, Milan Italy; 4 Azienda Ospedaliero-Universitaria Città della Salute e della Scienza, Turin, Italy; * These authors contributed equally to this work and shared first authorship

**Keywords:** Healthcare workers, vaccination adherence, vaccine preventable diseases, systematic review, meta-analysis

## Abstract

**Background:**

Vaccination adherence among healthcare workers (HCWs) is fundamental for the prevention of vaccine-preventable diseases (VPDs) in healthcare. This safeguards HCWs' well-being, prevents transmission of infections to vulnerable patients and contributes to public health.

**Aim:**

This systematic review and meta-analysis aimed to describe interventions meant to increase HCWs’ adherence to vaccination and estimate the effectiveness of these interventions.

**Methods:**

We searched literature in eight databases and performed manual searches in relevant journals and the reference lists of retrieved articles. The study population included any HCW with potential occupational exposure to VPDs. We included experimental and quasi-experimental studies presenting interventions aimed at increasing HCWs’ adherence to vaccination against VPDs. The post-intervention vaccination adherence rate was set as the main outcome. We included the effect of interventions in the random-effects and subgroup meta-analyses.

**Results:**

The systematic review considered 48 studies on influenza and Tdap vaccination from database and manual searches, and 43 were meta-analysed. A statistically significant, positive effect was seen in multi-component interventions in randomised controlled trials (relative risk (RR) = 1.37; 95% CI: 1.13–1.66) and in observational studies (RR = 1.43; 95% CI: 1.29–1.58). Vaccination adherence rate was higher in community care facilities (RR = 1.58; 95% CI: 1.49–1.68) than in hospitals (RR = 1.24; 95% CI: 0.76-2.05).

**Conclusion:**

Interventions aimed at increasing HCWs’ adherence to vaccination against VPDs are effective, especially multi-component ones. Future research should determine the most effective framework of interventions for each setting, using appropriate study design for their evaluation, and should compare intervention components to understand their contribution to the effectiveness.

## Introduction

Healthcare workers (HCWs) are at risk of contracting infectious disease at work and of transmitting infections to their patients, colleagues and families [1]. Indeed, HCWs are often sources of vaccine-preventable diseases (VPDs) in healthcare settings [1]. The World Health Organisation (WHO) estimates that ca 59 million HCWs worldwide are exposed to multiple occupational biohazards every day, through contact with infectious patients and with contaminated fluids and materials [2].

There are many benefits of vaccinating HCWs. Vaccination reduces the risk of infectious disease transmission within healthcare facilities and protect vulnerable patients [2]. Furthermore, vaccination can decrease illness and absenteeism among HCWs, which could lower the costs of healthcare services due to lost productivity [3]. Moreover, vaccinated HCWs are more likely to be knowledgeable about vaccinations and to be effective in improving confidence in vaccination among the general public [2,4]. Indeed, some studies have found that patients and their families consider vaccinated HCWs to be the most reliable sources of information regarding vaccinations, and a positive influence on their adherence to and trust in vaccinations [2,5,6]. During the COVID-19 pandemic, the involvement of HCWs in establishing functional vaccination programmes played a key role in facilitating timely vaccination adherence and containing the spread of the virus [7].

Vaccination adherence is generally defined as receiving a vaccination according to the vaccine’s label-recommended dose schedule [8]; it is a behaviour resulting from a decision-making process influenced by various determinants [9]. Within healthcare settings and among HCWs, vaccination adherence can exhibit notable differences [10,11]. It can be influenced by contextual immunisation programmes as well as policies, legal requirements, workplace and patients’ characteristics [2]. Despite long-standing recommendations that HCWs receive vaccinations against VPDs, their adherence to routine vaccination schedules is often suboptimal, leading to concern about preventing healthcare-associated infections [2,10,11]. European Union (EU) data about 2020/21 seasonal influenza vaccination showed that average vaccination coverage among HCWs is lower than the recommended vaccination coverage of 75% (median: 52%; range: 16–71) [2,10]. The hepatitis B vaccination rate in HCW in the EU is about 50% (update 2022), far lower than the recommended 80% [12]. The coverage with booster vaccinations for tetanus-diphtheria-acellular pertussis (Tdap) is variable worldwide, ranging from 6.1% to 63.9%, compared with the recommended 60% [13]. 

An HCW’s decision to get vaccinated involves a series of individual and social determinants [6,14,15]. Underestimation of the severity of disease and limited access to vaccination are among the major reasons for non-adherence among HCWs [5,14]. Furthermore, fear of side effects and misinformation or doubts about the effectiveness of vaccines seem to affect HCWs’ vaccination behaviours negatively [5,6,11]. Professional roles and work settings can also influence vaccination adherence in HCWs [15-17]. Low vaccination coverage is more frequent in healthcare assistants and nurses than in physicians, suggesting that vaccination adherence differs among HCWs professional categories. Those HCWs working in hospital settings generally have higher vaccination adherence [7,18]. Maybe vaccines are more easily accessible in hospitals than community settings. For instance, in contexts where vaccine shortages are prevalent, access to vaccinations in community settings can be more challenging [5,7,18].

Therefore, targeted interventions are needed to increase HCWs’ adherence to vaccination against VPDs. While systematic reviews have investigated determinants of HCWs’ adherence to vaccination against VPDs [19], there is little evidence on the effectiveness of interventions that aim to increase this adherence. Previous reviews or meta-analyses that assessed such interventions focused on seasonal influenza vaccination [20-23]; only one review evaluated Tdap vaccination among HCWs [24]. In particular, the systematic reviews by Rashid et al. [23], Hollmeyer et al. [20] and Lam et al. [21], as well as the meta-regression by Lytras et al. [22], classified interventions that aimed to increase seasonal influenza vaccination coverage among HCWs and evaluated their effectiveness. However, to the best of our knowledge, no recent studies have performed an overall evaluation of interventions that meant to increase HCWs’ adherence to vaccination against VPDs; instead, most existing reports cover the general population [25]. Also, no studies have tested the effectiveness of such interventions in subgroups of HCWs or healthcare settings. Furthermore, the literature is still fragmented and lacks a broad description of intervention characteristics, types, components, operational strategies and effectiveness. A better understanding of these could improve future vaccination campaigns and maximise healthcare resources.

The objective of this systematic review and meta-analysis was to describe interventions meant to increase HCWs’ adherence to vaccination against VPDs and to estimate the effectiveness of these interventions. More specifically, this study will provide evidence to answer the following research questions: (i) What interventions have been designed to increase HCWs’ adherence to vaccination against VPDs? (ii) What are the characteristics, types, components and operational strategies of these interventions? (iii) What was the effectiveness of each intervention? And (iv) are there any differences in effectiveness across subgroups of professional categories of HCWs or across healthcare settings?

## Methods

A systematic literature review and meta-analysis was conducted according to the updated Preferred Reporting Items for Systematic Review and Meta-Analyses (PRISMA) guidelines [26]; the PRISMA checklist is appended in the Supplement. The review protocol was registered in PROSPERO (CRD42020212252). In line with the original protocol, we included studies that evaluated vaccination adherence against VPDs by all HCWs.

### Search strategy

One investigator (BA) with experience carried out the literature searches under the supervision of a health librarian. To create an exhaustive search strategy and identify the most appropriate keywords, an explorative search of PubMed and CINAHL EBSCO was conducted in July 2022, followed by an analysis of the resultant titles and abstracts. Eight databases (PubMed, CINAHL EBSCO, Scopus, EMBASE, Web of Science, PsycInfo, The Cochrane Library and Joanna Briggs Institute) were searched from their beginning to 7 August 2022. Search strategies employed both thesaurus and free terms and were adapted for each database. The same author (BA) conducted manual searches in high-quality and relevant journals in the field – a list of which was taken from SCImago and Web of Science [27] – and on the reference lists of retrieved articles, to identify any additional relevant studies. A list of search terms is appended in the Supplement. No time limits were applied, and the search was limited to articles written in English and Italian.

### Eligibility and exclusion criteria

#### Studies were evaluated based on PICOS criteria

##### Population

We used the lists of disciplines developed by the WHO [28,29] and the international classification of HCWs [29] to determine which HCWs to include. To be eligible for inclusion, studies had to report information on HCWs from the disciplines of medicine (e.g. physicians, resident physicians), nursing (e.g. registered nurses, licensed practical nurses) or healthcare assistants, (e.g. healthcare unit assistants, nurse aids) who worked in healthcare settings and had the potential to be exposed to patients and/or to infectious materials. If authors reported on other disciplines (e.g. physiotherapists, radiology technicians, midwives), these disciplines were also considered. However, in studies that reported results from both HCWs and general staff (people who did not provide direct care to patients, e.g. cleaners, drivers, administrative staff and other disciplines) [28], we included only the HCWs in the review and meta-analysis.

##### Intervention

Studies of interventions aimed at increasing adherence, coverage, uptake or compliance to vaccination against VPDs among HCWs, in any setting or facility (i.e. public, private, teaching hospital, community care, home care) were eligible for inclusion. We excluded studies that described interventions that aimed to assess only HCWs’ knowledge, attitudes, vaccination intentions or behaviours, or interventions acting on the determinants of vaccination.

##### Comparator

We used an inactive control (e.g. standard vaccination practice, none).

##### Outcomes

We defined as outcome the post-intervention vaccination adherence rate, i.e. the proportion of HCWs reporting vaccination divided by the total study sample participating in the interventions.

##### Study design

Studies with experimental (e.g. randomised and non-randomised controlled trials) and quasi-experimental (hereafter referred to as observational, e.g. pre–post evaluation studies) designs were eligible for inclusion. Qualitative studies, protocols, proceedings, guidelines, discussion/editorials and reviews were excluded, but the reference lists of relevant reviews were screened for additional relevant articles that could be included here.

Recommended vaccinations for HCWs were defined as those put forth by the WHO [30] and considered vaccinations against diseases such as: diphtheria, *Haemophilus influenzae* type b infection, hepatitis A and B, influenza (seasonal), measles, meningococcal infections, mumps, pertussis, pneumococcal infections, poliomyelitis, rubella, tetanus and varicella (we chose to not include other recommended vaccinations such as for example Japanese encephalitis as these are not frequently encountered in Western countries). Furthermore, we excluded COVID-19 and human papillomavirus vaccines because COVID-19 specifically referred to the pandemic context and human papillomavirus vaccines are mainly targeted at a younger age group. In the case of articles with limited or unclear information on the PICOS criteria, the first or corresponding author was contacted by email. If no response was received, the article was excluded.

### Screening and data collection

The reference manager programme Endnote 20 [31] was used to collate articles identified in the databases and to identify duplicates. The titles and abstracts of remaining articles were then independently screened by two authors (BA and AC) using the automated tool Rayyan [32]. The full texts of studies that passed this screening were retrieved and assessed for eligibility. During this assessment, any disagreement or uncertainty regarding article inclusion was solved through consensus with a third author (MC). Data on study characteristics (design, setting, vaccination type and study period), participants, sample size, intervention type, components and operational strategies were extracted from included articles using a specifically designed data collection form; narrative descriptions for the meta-analysis were independently extracted by two authors (BA and AC). Disagreements and uncertainties were solved by consensus with a third researcher who is an expert in meta-analyses (MC). The data collection form was piloted on five studies, and appropriate adjustments were made before definitive data aggregation.

### Assessment of methodological quality and risk of bias

The methodological quality of included articles was independently assessed by two authors (BA, AC) using the Quality Assessment Tool for Controlled Intervention Studies and the Quality Assessment Tool for Before–After (Pre–Post) Studies with No Control Group developed by the National Heart, Lung, and Blood Institute [33]. The use of these two distinct tools ensured a comprehensive evaluation of study quality and allowed us to appropriately assess the quality of studies with different designs and methodologies. Each criterion was evaluated according to the tool’s questions, assigning cannot determine (CD), no (N), not reported (NR) or yes (Y). Based on this evaluation, the quality of included studies was rated as good (with only one CD, N, NR), fair (with two CD, N, NR) or poor (with more than two CD, N, NR). Any disagreement in the assessment of quality was solved through discussion with a third author (AC). Furthermore, two authors (BA, MC) assessed the risk of bias of included studies, using the Cochrane Risk-of-Bias tool for Randomised Trials (RoB 2) for randomised controlled trials (RCTs) [34] and the Risk of Bias In Non-Randomised Studies of Interventions (ROBINS-I) tool for observational studies [35]. These two instruments assessed the risk of bias in terms of randomness and concealment of subject allocation, blinding of subjects and outcome assessors, proportion of missing data, selective reporting and other sources of bias. The risk of bias for each domain was then categorised as low, some concerns or high for the RoB 2, and as low, moderate or high for ROBINS-I.

### Data overview and meta-analysis

The summary relative risk (RR) for the experimental group (or post-intervention timing in observational studies) as compared with the control group (or pre-intervention timing) was estimated using both the fixed-effects model and the random-effects model proposed by DerSimonian and Laird [36]. We estimated both 95% confidence intervals (CIs) and 95% prediction intervals [37]. When significant heterogeneity was found, we present the results from the random-effects model. The heterogeneity between study-specific estimates was measured with the I^2^ statistic [38]. Statistical significance level was set at a p value < 0.05.

We performed subgroup analyses to assess the effect that different intervention types (mono-component vs multi-component), professional categories (physicians, nurses, healthcare assistants and other HCWs) and healthcare settings (hospital, community care and facilities providing both hospital and community care services) had on post-intervention vaccination adherence rate. Sensitivity analyses were performed using the leave-one-out technique to control between-study heterogeneity [39]. A separate sensitivity analysis that excluded studies with a poor quality rating was also performed. Publication bias was evaluated through funnel plot inspection and the Egger’s test [40]. Analyses were performed using the statistical programme R with *metafor* and *meta* packages [39,41]. We performed a systematic narrative synthesis to present available data for all studies that could not be included in the meta-analyses.

## Results

**Figure 1:**
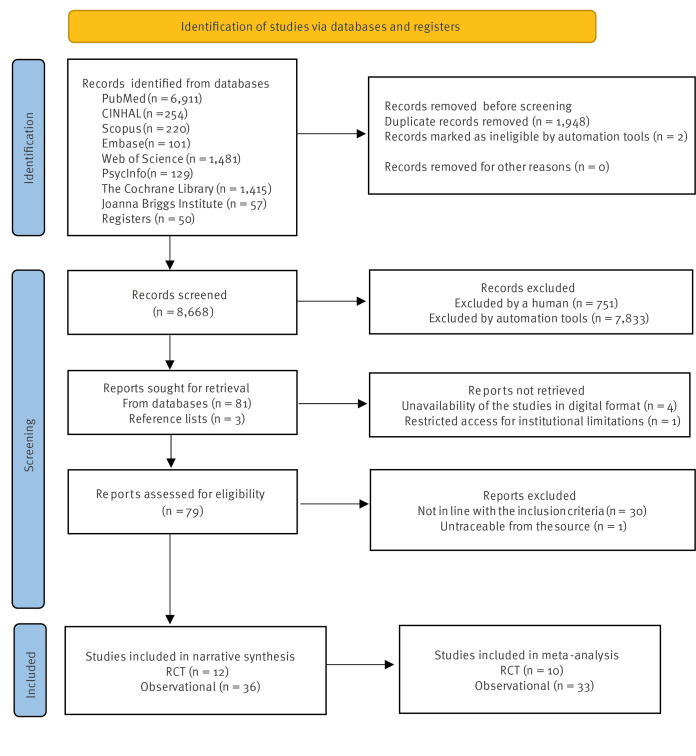
PRISMA flowchart, systematic review on effectiveness of interventions to increase healthcare workers’ adherence to vaccination

Search strategies identified a total of 10,618 articles (Figure 1). After the removal of duplicates, the title and abstract of 8,668 articles were screened, leading to the exclusion of 8,584 articles. This left 84 articles to be retrieved and their full texts assessed for eligibility. Five of these could not be retrieved and the authors could not be contacted, so 79 articles were finally assessed for eligibility. Of these 79 articles, 31 were excluded: thirty did not meet the inclusion criteria, and one was untraceable from any sources (Figure 1). After the full-text evaluation, 48 articles were included in the systematic review. Five of those articles [42-46] were not eligible for the meta-analysis, which finally included 43 articles [16-18,47-86].

### Study characteristics

The characteristics of the included studies are summarised in Table 1. The earliest study was reported in 1993 [46], with a peak in publication from 2014 to 2019 (n = 16). The studies were conducted in the EU (n = 25), mainly in France (n = 5) and Italy (n = 5), and in the US (n = 16), Canada (n = 2), Israel (n = 1), South Korea (n = 1) and Türkiye (n = 1). Twelve studies were RCTs [43,45,47,49,52,55,56,64,67,75-77], while 36 had an observational design [16-18,42,44,46,48,50,51,53,54,57-63,65,66,68-74,78-86]. Among the RCTs, three were cluster-randomised trials [49,67,77] and three had a 2 × 2 factorial design [43,45,64]. Randomisation in the RCTs was performed at the individual level [45,47,49,52,56,64,67] and at the unit (hospital/hospital ward) level [43,55,75-77]. Forty-four articles provided interventions for seasonal influenza vaccination [16-18,42-50,52-61,63-69,71-80,82-86], and four were on Tdap vaccination [51,62,70,81]. Studies recruited participants mainly in hospital settings (n = 30) [16,17,43,44,48,50,51,53-55,57-60,62,65,66,68,70,71,75-81,83,84,86] but 10 enrolled participants from community care [18,45-47,49,56,64,67,69,85]. The remaining studies were performed in facilities that provided both hospital and community care services (n = 8) [42,52,61,63,72-74,82]. Study periods ranged from 2 weeks [55] to 8 years [58].

**Table 1:** Characteristics of included studies on effectiveness of interventions to increase healthcare workers’ adherence to vaccination (n = 48)

Article (country, year)	Setting	Vaccination	Study period	Participants^a^	Sample size		Intervention
Randomised controlled trials (n = 12)					Intervention	Control	
Abramson [47] (Israel, 2010)	Community care	Seasonal influenza	2007–2008 (1 year)	Overall: 344 (physicians: 121; nurses: 68; other HCWs: 155)	163	181	Educational + promotional + policy
Borgey [49] (France, 2019)	Community care	Seasonal influenza	2014–2015 (1 year)	Overall: 1,336	496	840	Educational + promotional + policy
Chambers [52] (Canada, 2015)	Hospital and community	Seasonal influenza	2008–2012 (3 years)	Overall: 496,825^a^	165,079	331,746	Educational
Currat [55] (Switzerland, 2020)	Hospital	Seasonal influenza	2016 (2 weeks)	Overall: 357 (physicians: 116; nurses: 158; other HCWs: 83)	185	172	Educational
Dey [56] (UK, 2001)	Community care	Seasonal influenza	1999 (2 months)	Overall: 2,984 (physicians: 212; nurses: 1,531; healthcare assistants: 1,129; other HCWs: 112)	1,225	1,759	Educational + promotional
Golebiak [43]^b^ (Poland, 2020)	Hospital	Seasonal influenza	2017–2018 (1 year)	Overall: 327	245	82	Educational
Kimura [64] (US, 2007)	Community care	Seasonal influenza	2000–2002 (2 years)	Overall: 3,924 (nurses: 2,600; healthcare assistants: 1,000; other HCWs: 324)	2,407	1,517	Educational + promotional
Looijmans-van den Akker [67] (the Netherlands, 2010)	Community care	Seasonal influenza	2006 (1 year)	Overall: 6,608 (physicians: 733; nurses: 1,054; healthcare assistants: 4,821)	3,058	3,550	Educational + promotional
Rothan – Tondeur [75] (France, 2010)	Hospital	Seasonal influenza	2005–2006 (2 years)	Overall: 2,345 (physicians: 153; nurses: 586; healthcare assistants: 1,115: other HCWs: 491)	1,201	1,144	Educational
Rothan – Tondeur [76] (France, 2011)	Hospital	Seasonal influenza	2006–2007 (2 years)	Overall: 2,874 (physicians: 302; nurses: 686; healthcare assistants: 1,506: other HCWs: 380)	1,335	1,539	Educational
Saunier [77] (France, 2020)	Hospital	Seasonal influenza	2018–2019 (1 year)	Overall: 3,547	1,968	1,579	Informative
Schmidtke [45]^b^ (UK, 2020)	Community care	Seasonal influenza	2019–2020 (1 year)	Overall: 8,438 (physicians: 1,440; nurses: 4,478; healthcare assistants: 2,520)	7,540	898	Educational + promotional + policy
Observational (n = 36)					Vaccinated after intervention	Vaccinated before intervention	ׅ
Babcock [42]^b^ (US, 2010)	Hospital and community	Seasonal influenza	2008–2009 (1 year)	Overall: NS	25,561	25,980	Educational + promotional + policy
Bert [48] (Italy, 2019)	Hospital	Seasonal influenza	2017–2018 (1 year)	Overall: 1,186	503	373	Educational + promotional
Boey [18] (Belgium, 2021)	Community care	Seasonal influenza	2016–2017 (1 year)	Overall: 828	368	378	Educational + promotional
Butteri [50] (US, 2010)	Hospital	Seasonal influenza	2007–2008 (1 year)	Overall: 106	77	69	Educational
Calderon [51] (US, 2008)	Hospital	Tdap	2007–2008 (1 year)	Overall: 1,281	484	447	Educational + promotional
Chittaro [53] (Italy, 2009)	Hospital	Seasonal influenza	2004–2006 (2 years)	Overall: 473 (physicians: 106; nurses: 254; healthcare assistants: 113)	283	49	Educational + promotional
Conte [84] (Italy, 2016)	Hospital	Seasonal influenza	2013–2014 (1 year)	Overall: 881 (physicians: 251; nurses: 423; healthcare assistants: 157; other HCWs: 50)	221	164	Educational + promotional
Cozza [54] (Italy, 2015)	Hospital	Seasonal influenza	2009–2013 (5 years)	Overall: 792	35	162	Educational + promotional
de Juanes [57] (Spain, 2007)	Hospital	Seasonal influenza	2002 – 2004 (2 years)	Overall: 5,654 (physicians: 1,177; nurses: 1,758; healthcare assistants: 2,719)	3,449	905	Educational + promotional
Frenzel [58] (US, 2016)	Hospital	Seasonal influenza	2006–2014 (8 years)	Overall: 34,807	17,927	8,762	Educational + promotional + policy
Frisina [59] (US, 2019)	Hospital	Seasonal influenza	2014–2018 (4 years)	Overall: 102	92	72	Educational + promotional
Gilardi [60] (Italy, 2018)	Hospital	Seasonal influenza	2016–2018 (2 years)	Overall: 4,254 (physicians: 1,044; nurses: 2,133; healthcare assistants: 1,077)	369	278	Educational + promotional
Heinrich – Morrison [61] (Australia, 2015)	Hospital and community	Seasonal influenza	2013–2014 (1 year)	Overall: 14,359 (physicians: 2,188; nurses: 6,517; healthcare assistants: 2,646; other HCWs: 3,008)	6,009	3,866	Educational + promotional
Jiang [62] (US, 2018)	Hospital	Tdap	2014–2015 (1 year)	Overall: 1,997 (physicians: 747; nurses: 1,050; other HCWs: 200)	911	571	Educational + promotional
Kim [63] (US, 2018)	Hospital and community	Seasonal influenza	2011–2013 (3 years)	Overall: 690	301	240	Educational + promotional
Kuntz [65] (US, 2008)	Hospital	Seasonal influenza	2003–2006 (3 years)	Overall: 26,959	6,539	5,741	Educational + promotional
Leitmeyer [66] (Germany, 2006)	Hospital	Seasonal influenza	2002–2004 (2 years)	Overall: 792 (physicians: 306; nurses: 286; other HCWs: 200)	103	83	Educational + promotional + policy
LIupìa [83] (Spain, 2010)	Hospital	Seasonal influenza	2007–2009 (2 years)	Overall: 9,632 (physicians: 2,127; nurses: 2,867; healthcare assistants: 1,429; other HCWs: 3,209)	1,769	1,091	Educational + promotional + policy
Marwaha [68] (Canada, 2016)	Hospital	Seasonal influenza	2013 (1 year)	Overall: 19,398	2,062	2,439	Educational + promotional
Nace [69] (US, 2011)	Community care	Seasonal influenza	2002–2003 (1 year)	Overall: 2,550	485	432	Educational + promotional
Ofstead [85] (US, 2017)	Community care	Seasonal influenza	2014–2015 (1 year)	Overall: 2,732	640	726	Educational + promotional
Oguz [16] (Türkiye, 2019)	Hospital	Seasonal influenza	2016–2018 (2 years)	Overall: 1,144 (physicians: 403; nurses: 386; healthcare assistants: 355)	228	62	Policy
Paranthaman [70] (UK, 2016)	Hospital	Tdap	2012 (1 year)	Overall: 553	121	389	Educational + promotional
Podczervinski [71] (US, 2015)	Hospital	Seasonal influenza	2010–2013 (3 years)	Overall: 3,087	1,583	1,264	educational + promotional + policy
Qureshi [44]^b^ (UK, 2004)	Hospital	Seasonal influenza	2000–2001 (1 year)	Overall: 530 (physicians: 31; nurses: 290; healthcare assistants: 35; other HCWs: 158; not specified: 16)	514	NS	Educational + promotional
Rakita [72] (US, 2010)	Hospital and community	Seasonal influenza	2005–2010 (5 years)	Overall: 9,727	4,967	4,588	Educational + promotional + policy
Ribner [73] (US, 2008)	Hospital and community	Seasonal influenza	2006–2007 (1 year)	Overall: 18,264	6,123	3,892	Educational + promotional + policy
Rodriguez-Fernandez [74] (Spain, 2016)	Hospital and community	Seasonal influenza	2012–2013 (1 year)	Overall: 685 (physicians: 190; nurses: 294; healthcare assistants: 201)	137	105	Educational
Sartor [17] (France, 2004)	Hospital	Seasonal influenza	2000–2002 (2 years)	Overall: 2,512 (physicians: 396; nurses: 707; other HCWs: 1,409)	654	587	Educational + promotional
Smedley [78] (UK, 2002)	Hospital	Seasonal influenza	1998–1999 (1 year)	Overall: 13,268 (physicians: 1,564; nurses: 5,947; healthcare assistants: 866; other HCWs: 4,891)	292	156	Educational + promotional
Smithers [79] (Australia, 2003)	Hospital	Seasonal influenza	2000–2001 (1 year)	Overall: 541 (physicians: 161; nurses: 283; other HCWs: 97)	65	51	Policy
Song [86] (Korea, 2006)	Hospital	Seasonal influenza	2013–2014 (1 year)	Overall: 2,227 (physicians: 1,020; nurses: 1,000; other HCWs: 207)	882	253	Educational + promotional
Tapiainen [80] (Switzerland, 2005)	Hospital	Seasonal influenza	2003–2005 (2 years)	Overall: 1,092 (physicians: 193; nurses: 643; other HCWs: 256)	133	100	Educational + promotional
Thomas [46]^b^ (US, 1993)	Community care	Seasonal influenza	1990–1991 (1 year)	Overall: 868 (physicians: 101; nurses: 200; other HCWs: 567)	54	46	Educational + promotional + policy
Walther [81] (Switzerland, 2015)	Hospital	Tdap	2012–2013 (1 year)	Overall: 854 (physicians: 186; nurses: 525; other HCWs: 143)	304	72	Educational + promotional
Zimmerman [82] (US, 2009)	Hospital and community	Seasonal influenza	2005–2007 (2 years)	Overall: 53,668	10,784	8,565	Educational + promotional

NS: not specified; Tdap: tetanus, diphtheria, pertussis; UK: United Kingdom; US: United States.

^a ^Overall means overall sample of the study. For RCTs, ‘Overall’ is the sum of cases and controls. For observational studies, ‘Overall’ is the overall sample of the study (composed by both vaccinated and unvaccinated before and after intervention).

^b ^Article not meta-analysed.

### Participants

A total of 768,402 HCWs were included in the 48 studies. Sample sizes ranged from 327 [43] to 496,825 [52] in RCTs, and from 102 [59] to 51,541 [42] in observational studies. Twenty-six studies [16,17,44-47,53,55-57,60-62,64,66,67,74-76,78-81,83,84,86] specified the number of participating HCWs: physicians accounted for 15,268 participants, nurses for 36,724 and healthcare assistants for 21,689. There were 15,940 participants who fell into the category of other HCWs, including rehabilitation technicians, pharmacists and laboratory staff. Twenty-two articles did not specify the number or professional category of HCWs [14,42,43,48-52,54,58,59,63,65,68-73,77,82,85]. The age of participants ranged from 18 to 66 years. The majority of HCWs were female (n = 55,310); males accounted for 4,202 participants. Only four articles [43,54,60,86] considered HCWs’ duration of employment, with a minimum of 2 years [60] and a maximum over 40 years [43].

### Interventions

The components of interventions were classified as informational when they involved the distribution of instructive or decision-aid tools such as leaflets or booklets which provided essential evidence-based information about VPDs and vaccines or facilitated well-informed decision making of HCWs. They were defined as promotional when the interventions included active promotion of information or education on vaccines and vaccinations through broad campaigns that included operational strategies such as communicational or promotional activities. Components were defined as educational when the intervention aimed to change HCWs’ knowledge and/or attitudes, or applied specific educational methodologies or strategies such as role playing or video-modelling. Lastly, components were classified as policy when the interventions proposed programmes, activities or mandated actions by regulatory authorities or healthcare organisations.

Based on the number of components, interventions were organised into two overarching types: mono-component (containing one component, e.g. an educational component only) and multi-component interventions (containing two or more components, e.g. an educational and a promotional component). The majority of studies investigated multi-component interventions (n = 38) [17,18,42,44-49,51,53,54,56-73,78,80-86]. Among these, 27 used a combination of educational and promotional components, and 11 used a combination of educational, promotional and policy components. Ten studies [16,43,50,52,55,74-77,79] used mono-component interventions such as an educational component (n = 7), a policy component (n = 2) or an informational component (n = 1) alone. The operational strategies applied for each component were organised by contents, modalities of intervention delivering, tools employed and the frequency and number of sessions that the intervention comprised (all components and interventions for each study are detailed in the Supplement).

#### Mono-component interventions

##### Informational

Saunier et al. [77] investigated an intervention with an informational component in which an informative leaflet and a decision aid tool on seasonal influenza vaccination was distributed to HCWs. The leaflet listed five bullet points which contained information on seasonal influenza virus, its principal transmission route, the possible option to decrease the risk of getting seasonal influenza, and the possible benefits of vaccinations. One thousand leaflets were distributed during an in-service information session.

##### Educational

The contents of interventions with an educational component varied across studies [43,50,52,55,74-76]. Most contents were related to virus transmission channels, immunology and virology, and misconceptions about vaccination. The modalities principally included in-service workshops or theoretical lessons about vaccines and vaccinations. For example, Chambers et al. [52] and Rothan-Tondeour [75,76] proposed active educational in-service learning, while Golebiak et al. [43] proposed a training on influenza and a follow-up meeting after 2 weeks with educators. PowerPoint presentations and leaflets were the most frequently used educational tools. The duration of the educational sessions varied from 5 min per week [55] to 2 h per week [75,76]

##### Policy

Interventions with a policy component provided operational strategies to increase HCWs adherence to vaccination against VPDs [16,79]. No details were provided on the content of these interventions. The interventions were delivered following the policies on seasonal influenza immunisation implementation in their local contexts. Specifically, we have chosen to categorise as policy interventions those improving vaccine accessibility for HCWs and providing data for the ongoing evaluation and enhancement of vaccination policies. In particular, the intervention provided by Oguz et al. [16] used a mobile immunisation team to visit and immunise on-site HCWs in a hospital setting, and collected information about previous vaccination uptake.

#### Multi-component interventions

##### Educational and promotional

Two of the multi-component interventions were based on conceptual or mapping models [67,85]. In particular, the included educational components offered a variety of theoretical contents mainly related to immunology, misconceptions on immunisation, and infectious preventive measures. The modalities of delivery for the educational components were in-service lectures, discussions, and online learning. The teaching tools used were videos featuring role models, video recordings of lectures, and slideshows. The educational components included sessions that lasted anywhere from 2 min (e.g. online educational videos) to 6 h (e.g. lectures or plenary discussions) with intervals of 3 weeks to 2 months between sessions. Furthermore, the promotional components described in Chittaro et al. [53], Kuntz et al. [65] and Dey et al. [56] included free immunisation and activities that promoted vaccination. The most used promotional tools were informational websites, leaflets and posters. The promotional components in the intervention reported by Podczervinski et al. [71] used in-service coordinators to distribute materials and monitor promotional activities. Ribner et al. [73] used a task force to constantly verify vaccination trends and ensure consistent timely communication. The duration, frequency and sessions of these promotional activities were not specified.

##### Educational, promotional and policy

Two of the multi-component interventions were based on conceptual multi-phase models: the intervention of Borgey et al. [49] was based on the Five Keys WHO model, and that of Schmidtke et al. [45] was based on the nudge theory. In particular, the nudge theory involves influencing behaviour through subtle suggestions or environmental modifications without coercion [87]. The contents of the educational components were mostly related to HCWs’ misconceptions about vaccination against VPDs and strategies to prevent infection; only two interventions [42,72] had educational components based on theoretical content that referred to immunology and virology. Teaching methods included in-service education and training, and the most common educational tools were slideshows, role-playing and clinical case discussions. Some interventions created a multidisciplinary team (e.g. nurses, physicians, psychologists and managers) to achieve a more effective management of information and communication channels. In particular, the intervention reported by Babcock [42] organised ‘town hall meetings’ during a vaccination campaign, during which HCWs received vaccination training. The duration of educational activities varied from 1 h (e.g. on-site training) [61] to 6 h per session (e.g. lectures or discussions) [53]; the intervention of Looijmans-van den Akker et al. [67] offered two sessions of 1 h each.

The most frequently used tools for promotional components were posters, leaflets, internal newspapers, email newsletters and websites. Babcock et al. [42] created a dedicated question and answer blog concerning vaccinations. The policy components included recommendations and guidelines for vaccination monitoring, which generally referred to other institutional sites (e.g. WHO). Some interventions used mandatory vaccination or financial compensation to increase HCWs’ interest in getting vaccinations. In particular, Frenzel et al. [58], Ribner et al. [73] and Walther et al. [81] each implemented distinct mandatory activities within their prevention programmes. Frenzel et al. [58] employed mandatory vaccinations, Walther et al. [81] organised mandatory appointments for administering vaccinations, and Ribner et al. [73] introduced a mandatory declination form for those opting to refuse vaccination, while Calderon et al. [51], Leitmeyer et al. [66] and LIupià et al. [83] used monetary compensation or gifts as a part of their intervention to increase HCWs’ vaccination adherence. However, none of the studies specified the duration of the promotional or policy components of interventions.

### Study quality and risk of bias

The overall quality of evidence was evenly distributed for RCTs: four articles had a rating of good quality [49,55,67,77], four had a rating of fair quality [47,52,64,75] and four had a rating of poor quality [43,45,56,76]. None of the observational studies had a rating of good quality. Instead, 24 had a rating of fair quality [16-18,42,48,53,54,57-60,62,69-71,73,74,78-80,83-86] and 11 had a rating of poor quality [44,46,50,51,58,65,66,68,72,81,82]. All details about quality assessment are appended in the Supplement. 

**Figure 2:**
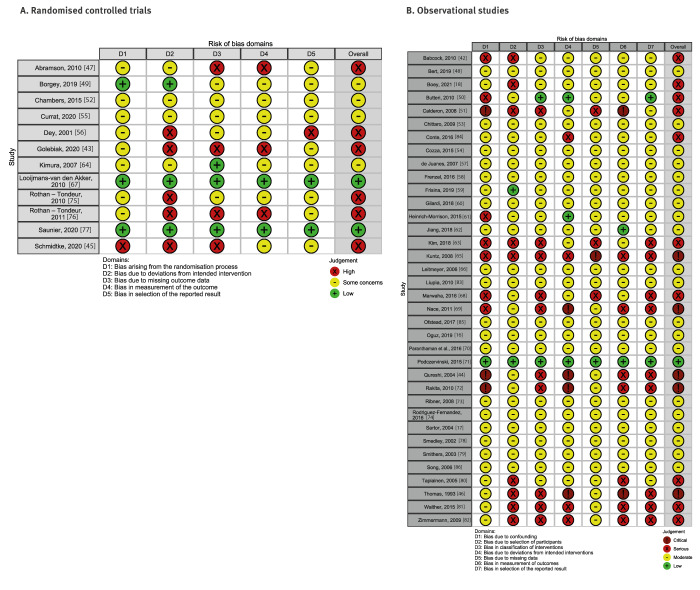
Study quality and risk of bias of publications on effectiveness of interventions to increase healthcare workers’ adherence to vaccination (n = 48)

The risk of bias was high for the half of the RCTs (n = 6) [43,45,47,56,75,76] (Figure 2); four RCTs presented some concerns of bias [49,52,55,64]; and only two had a low risk of bias [67,77]. All the observational studies were found to have a low or moderate risk of bias. The most problematic issues were confounding, measurement of outcomes bias and bias in the selection of reported results. Moderate bias due to confounding was found in all the included observational studies. Twenty-five had moderate bias in the measurement of outcomes [16,17,42,48,50,53,54,57-63,66,68,70,73,74,78,79,83-86], and 25 showed moderate bias in the selection of reported results [16-18,42,48,51,53,54,57-62,66,70,73,74,78-80,83-86] (Figure 2).

### Random-effects meta-analysis stratified by vaccination and type of intervention

#### Vaccine-preventable diseases

**Figure 3:**
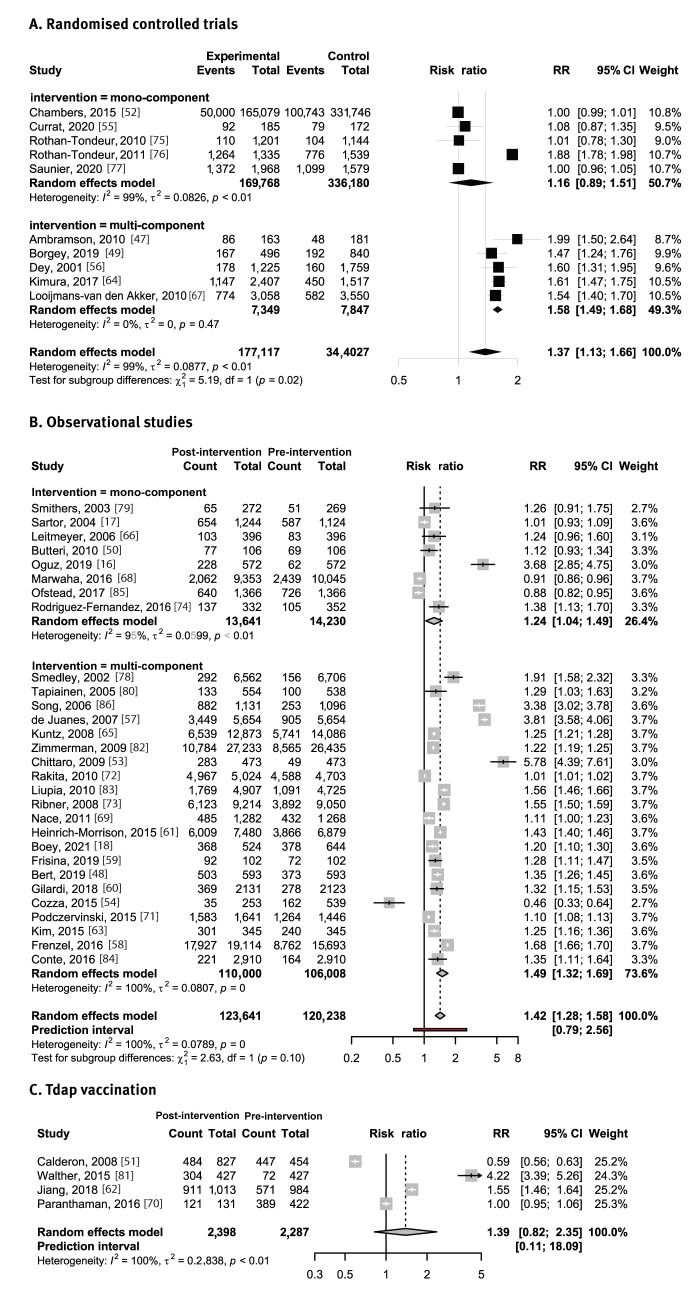
Plots of random-effects meta-analysis stratified by vaccination and type of intervention for publications on effectiveness of interventions to increase healthcare workers’ adherence to vaccination (n = 43)

CI: confidence interval; RR: relative risk; Tdap: tetanus-diphtheria-acellular pertussis.

Figure 3 shows the effect of mono-component and multi-component interventions on HCWs’ adherence to vaccination against VPDs in RCTs and observational studies. In RCTs, the implementation of multi-component interventions yielded a higher, statistically significant, positive effect (RR = 1.58; 95% CI: 1.49–1.68) on HCWs’ post-intervention adherence to seasonal influenza vaccination than did mono-component interventions (RR = 1.16; 95% CI: 0.89–1.51). Moreover, in observational studies, multi-component interventions showed a higher, statistically significant, positive effect on post-intervention adherence to seasonal influenza vaccination (RR = 1.49; 95% CI: 1.32–1.69) when compared with mono-component interventions (RR = 1.24; 95% CI: 1.04–1.49). Multi-component interventions also showed a positive effect on post-intervention adherence to Tdap vaccinations (RR = 1.39; 95% CI: 0.82–2.35), but this increase was not statistically significant (Figure 3, Panel C).

#### Subgroup meta-analysis by professional category

The interventions described in RCTs led to a statistically significant increase in post-intervention adherence to seasonal influenza vaccination in all professional categories, with a larger increase among nurses (RR = 1.59; 95% CI: 1.06–2.40) than healthcare assistants (RR = 1.41; 95% CI: 1.06–1.87) and physicians (RR = 1.37; 95% CI: 1.13–1.66). Nevertheless, the difference between professional categories was not statistically significant (p = 0.84).

In observational studies, all professional categories presented a statistically significant increase in post-intervention vaccination adherence rate, with a higher increase among healthcare assistants (RR = 1.64; 95% CI: 1.22–2.19) than other professional categories (RR = 1.43; 95% CI: 1.07–1.90), but the differences between groups were not statistically significant (p = 0.79) (Table 2).

**Table 2:** Sub-group meta-analyses of interventions to increase healthcare workers’ adherence to vaccination, by category of work and setting (n = 43 interventions)

	Number of studies	RR	95% CI
**Randomised controlled trials**			
**HCW category**			
Physicians	4	1.37	1.13–1.66
Nurses	4	1.59	1.06–2.40
Healthcare assistants	3	1.41	1.06–1.87
Others	2	1.29	1.02–1.62
Random effects		1.43	1.25–1.64
I^2^	79% (p < 0.01)		
χ^2^	0.84 (p = 0.84)		
Settings			
**Hospital**	3	1.24	0.76–2.05
Community care	5	1.58	1.49–1.68
Hospital and community care	2	1.00	0.99–1.01
Random effects		1.37	1.25–1.64
I^2^	99% (p < 0.01)		
χ^2^	246.05 (p < 0.01)		
**Observational studies**			
**HCW category**			
Physicians	13	1.40	1.20–1.63
Nurses	13	1.38	1.16–1.64
Healthcare assistants	8	1.64	1.22–2.19
Others	7	1.43	1.07–1.90
Random effects		1.43	1.31–1.56
I^2^	94% (p < 0.01)		
χ^2^	1.07 (p = 0.79)		
Settings			
Hospital	22	1.50	1.32–1.70
Community care	3	1.05	0.87–1.28
Hospital and community care	4	1.40	1.29–1.51
Random effects		1.43	1.29–1.58
I^2^	100% (p < 0.01)		
χ^2^	9.11 (p = 0.01)		

CI: confidence interval; HCW: healthcare worker; RR: relative risk.

#### Subgroup meta-analysis by healthcare setting

In the RCTs carried out in community care settings, interventions had a positive effect and were followed by a statistically significant increase in post-intervention adherence to vaccination against seasonal influenza (RR = 1.58; 95% CI: 1.49–1.68). We found no relation in HCWs' post-intervention adherence to vaccination against seasonal influenza in hospital settings (RR = 1.24; 95% CI: 0.76–2.05) (Table 2).

In observational studies set in hospitals, interventions had a positive effect on HCWs’ post-intervention vaccination adherence rate (RR = 1.50; 95% CI: 1.32–1.70). In studies set in facilities providing both hospital and community care (RR = 1.40; 95% CI: 1.29–1.51), we found a not statistically significant effect in community care (RR = 1.05; 95% CI: 0.87–1.28) (Table 2).

### Publication bias and sensitivity analysis

No publication bias was seen for RCTs or observational studies on seasonal influenza (p = 0.150 and p = 0.057, respectively) and for Tdap vaccination (p = 0.570).

For seasonal influenza, the leave-one-out sensitivity analysis did not modify the results, neither for RCTs nor for observational studies. However, for Tdap vaccination, the removal of the studies with extreme results [51,81] did modify the results of the pooled random effects model. We also performed a sensitivity analysis after removing studies with poor quality; it did not reveal any differences in the results for seasonal influenza vaccination in RCTs or observational studies, but made the pooled RR statistically significant for observational studies on Tdap vaccination (RR = 1.84; 95% CI: 1.14–2.97). All details about publication bias and sensitivity analysis are appended in the Supplement.

## Discussion

This systematic review and meta-analysis proved that interventions aimed at increasing vaccination adherence for influenza and Tdap vaccines among HCWs are effective, especially multi-component ones. Unlike a previous work [22], this review performed meta-analyses stratified by type of intervention and performed subgroup meta-analyses by professional category and healthcare setting. Interventions were more effective in nurses and healthcare assistants than in other HCW groups. According to the observational studies, interventions in community care settings were less effective than those performed in hospital settings and in facilities including both hospital and community services. Conversely, RCTs showed that interventions in hospital settings were not significantly different from those carried out in community care settings.

Our results show that most studies on the effectiveness of interventions are observational; observational or descriptive methodologies are generally preferred to RCTs to measure the effects of vaccination programmes or to increase vaccination coverage on a population level [88]. Indeed, observational studies can include much larger populations than RCTs [89]. Although observational studies are less precise (e.g. wider CIs) and more prone to bias, they may be more practical (e.g. shorter duration), and they ensure external validity and generalisability of the data [89].

The interventions that met the inclusion criteria of this systematic review related to influenza and Tdap vaccinations. In the US, the latest data on the rate of seasonal influenza vaccination among HCWs showed that the COVID-19 pandemic increased HCWs’ adherence to seasonal influenza vaccination, but uptake was still limited [90]. However, this result is not entirely generalisable worldwide. Recent data [91] showed a significant increase in seasonal influenza vaccination rates among HCWs during and immediately after the COVID-19 pandemic. Therefore, the pandemic seemed a major factor in HCWs’ adherence to vaccination, to protect families and patients, and demonstrated moral and civic responsibility. Our meta-analysis suggested that interventions do affect HCWs’ adherence to influenza vaccination, with vaccination adherence increased by 43% in observational studies and 37% in RCTs. An increase in vaccination adherence by at least 35% is recognised as the minimum required to yield a potential protective impact on acquired influenza infection [3,92].

Tetanus and diphtheria are rare diseases in high-income countries, and Tdap vaccination policies during childhood vary globally [13]. In many high-income countries, Tdap vaccination is mandatory during childhood, but this is not the case in all regions. Despite these differences, Tdap vaccinations are strongly recommended for HCWs across various settings [92]. The US Centers for Disease Control and Prevention recommended that HCWs receive a Tdap booster every 10 years [12], but a recent review [93] showed that HCWs’ adherence to Tdap boosters was under 40% and pertussis outbreaks are increasing, especially in neonatology, paediatrics and obstetrics units. Results from our meta-analysis are in line with previous evidence, as it seemed that interventions were not significantly effective in increasing HCWs’ adherence to Tdap boosters.

Subgroup meta-analyses showed the positive effect of multi-component interventions on HCWs’ adherence to vaccination against VPD such as influenza and Tdap. There is consensus that mono-component interventions are not effective in increasing vaccination adherence among HCWs rapidly and substantially [22]. Indeed, contextual, psychological and social factors and determinants act on an individual’s interest and behaviour towards vaccination [19,94]. Therefore, multi-component interventions, by better addressing these underlying behavioural complexities, would effectively encourage vaccination adherence among HCWs [19,95]. The literature states that successful interventions should contain as many components as possible [20,22]. However, in resource-limited settings, interventions could focus on at least two components to rapidly increase vaccination rates, such as educational and promotional components [21,22]. The combination of educational and promotional components in particular seemed to increase HCWs’ vaccination adherence [21]. Moreover, interventions delivered as in-ward training combined with promotional campaigns had a positive effect on HCWs’ vaccination adherence, increasing it by almost 20% [20,95]. Furthermore, educational initiatives delivered as in-practice training in parallel with the distribution of informative materials increased HCWs’ adherence, their level of knowledge, and led to positive attitudes towards vaccination [96]. The Strategic Advisory Group of Experts on Immunization (SAGE) highlighted the importance of finding a balance in planning vaccination interventions and advises that before tailoring specific interventions, it is essential to first assess the root causes affecting vaccine uptake [97,98]. The diverse needs and contexts of HCWs must be considered when designing effective strategies. When promotional strategies are poorly implemented, HCWs may be more reluctant to vaccinate, while clear, accurate and strong promotional or communicational activities improve HCWs’ awareness of vaccination [97,98]. The review by Jarret et al. [19] reported that theoretical education combined with strong promotional activities increased HCWs’ adherence to vaccination by more than 25%. Furthermore, a strong promotional campaign improved HCWs’ trust in vaccinations [99]. 

Policy amendments (e.g. free vaccination), were effective in increasing adherence to seasonal influenza vaccination [22,23] and multi-component interventions that included free access to vaccination increased HCWs’ post-intervention vaccination adherence by 30% [19,22]. However, the effectiveness of such interventions depends on the type of policy. Mandatory or financial policy strategies could be quite effective on their own, but the diffusion of recommendations or guidelines on vaccination did not achieve the same positive results [21,22], especially when they were not applied continuously and for a long time. Educational materials or guidelines on vaccination need to be distributed continuously for at least 1 year, and the intervention should be based on an implementation protocol of ca 2 years, to ensure continuity in vaccination adherence among HCWs [2]. Indeed, prolonged interventions seem to increase HCWs’ vaccination adherence by more than 90% [100]. Previous studies have shown that mandatory workplace vaccination policies or financial incentives also increase HCWs’ vaccination uptake, especially during the COVID-19 pandemic [101] or for seasonal vaccinations [22]. However, there are ethical issues to these policies, as they can be seen as morally coercive [102]. Multi-component interventions which applied soft policy strategies that consider the local context and facilitate access to vaccination have been more effective than strong, mandatory policies [99].

It is of the utmost importance that interventions be based on a conceptual model or framework that supports the effectiveness of the interventions [3,21,89]. Despite the availability of taxonomies or classification models such as the SAGE model [98], behavioural models, situational behavioural theories and health belief models that are common in the public health literature on vaccination, such as during the recent COVID-19 pandemic [103], conceptual models and frameworks are still infrequently used in the construction of interventions. Conceptual models or theories allow researchers to identify predictors of individual vaccination behaviour and the barriers or facilitators on which they can intervene to promote vaccination [103]. The WHO recommends the use of conceptual models when planning interventions on vaccination, and the five-factors model [104] was recommended by the European Centre for Disease Prevention and Control during the COVID-19 pandemic. This model groups vaccination determinants to facilitate vaccination acceptance and has been adopted as the standard for implementing interventions to promote vaccines [105]. Further models exist to facilitate vaccination readiness and acceptance, such as the 7C model [106,107] or the ProVac-ce model [108], useful in assessing HCWs' psychosocial determinants towards vaccination. Recently, the WHO has adopted the COM-B model as a framework for implementing targeted vaccination interventions [109,110]. It could be a possible solution in designing targeted interventions for HCWs, as it provides a comprehensive approach to understanding and influencing behaviour in the context of vaccination. Surprisingly, the interventions found in our systematic review rarely used this conceptual model, and none of the studies applied it to plan their interventions.

Our meta-analysis showed the positive effects of interventions on vaccination adherence by professional category, especially in HCWs commonly defined as more hesitant, such as nurses and healthcare assistants [111]. Indeed, vaccination coverage is lower among nurses and healthcare assistants than physicians, and healthcare assistants tend to have a more negative attitude towards vaccination than nurses and physicians [112]. Nurses’ or healthcare assistants’ hesitancy may be stem from a lack of knowledge on vaccines (e.g. different types of vaccines or recommendations), or from a belief that VPDs are minor illnesses [112,113]. 

Contrasting evidence emerged from the subgroup meta-analysis by healthcare setting. Interventions proposed in observational studies were more effective in increasing vaccination adherence in hospital settings than in community care settings. In contrast, interventions proposed in RCTs obtained a more positive effect in community care settings. It is likely that the characteristics of community care settings (e.g. smaller or more balanced populations) allowed researchers to have better study control, avoid bias or pay greater attention to inclusion and exclusion criteria [114]. It is plausible to assume that conducting a hospital RCT on a topic related to patient safety (and not only to operator safety) could be difficult, especially with regard to the ethical requirements of trials, so in this setting, observational studies are probably easier to do than RCT [114,115]. However, further high-quality RCTs are needed in both settings to prevent methodological biases, a point that has been underlined in earlier reviews [116].

The results of this meta-analysis are affected by a large amount of heterogeneity, which could be caused by the clinical, methodological and statistical differences in the primary studies [117]. Firstly, the individual components of interventions were often very different, and the samples of HCWs were not homogenous: some studies interviewed staff from a single professional category, whereas the majority pooled different HCWs of different professional categories using a convenience-based sampling method. Furthermore, the different healthcare settings, countries (culture may affect intervention effectiveness), the specific details of each intervention, and the way these interventions were implemented could not be homogeneous.

When the methodological quality of studies included in a meta-analysis is good, it is expected to confer a lower risk of bias and yield reliable results [34,35]. It is well known that when small (and underpowered) studies are included in a meta-analysis, this tends to render more extreme treatment effects than when larger studies are included [118]. In our meta-analysis, this issue was evident in the assessment of adherence to Tdap vaccination, as the poor quality of the included studies affected and reduced the pooled effect of the interventions. It is possible that individual quality measures (e.g. anonymisation or allocation concealment) were not consistently considered in the included studies.

The main limitation of this systematic review and meta-analysis lies in the overrepresentation of influenza vaccination, with only few studies addressing Tdap and none addressing other vaccines such as measles, mumps, rubella. This limits both the generalisability of our findings to other VPDs and the possibility of conducting more in-depth analyses. Therefore, future research should provide a more comprehensive understanding of interventions on HCWs' adherence to specific vaccinations. The poor quality of some of the included studies negatively affected the power of the meta-analytic results. The different demographic characteristics of participants and the various HCW groups and settings considered led to high heterogeneity across results. Furthermore, no assessment was possible about the mandatory nature of vaccinations according to different settings or about the possible choice of getting the vaccine, as this information was not available for all studies. Almost none of the participants in RCTs were masked in terms of treatment allocation, given the nature of the intervention itself. Although search syntaxes were conducted with the assistance of an expert librarian, the sensitivity of the search was highly dependent on the specific database used, which might have led to missing some studies. It is important to note that updates in the literature were carefully monitored, with the latest screening conducted in 2022. 

## Conclusions

Multi-component interventions had a greater positive effect than mono-component interventions, and we saw the effectiveness of interventions on several categories of HCWs. This meta-analysis pointed out which interventions could be useful to promote higher vaccination adherence (for influenza and Tdap vaccinations) in this population. In particular, multi-component interventions using suitable components and operational strategies could increase and promote vaccination adherence among HCWs and support vaccination policies. Therefore, our results could enhance evidence-based policymaking and maximise healthcare resources. Future research should guide decision-makers in determining the most effective frameworks for the implementation of interventions, the most reliable study design in specific settings (e.g. RCTs for community care vs observational for hospital settings), and in comparing different components to determine how they contribute to the effectiveness of interventions. 

## Supplementary Data

1722000 Supplement

## References

[1] Occupational Safety and Health Administration (OSHA). Healthcare. Washington: OSHA. [Accessed: 23 Feb 2023]. Available from: https://www.osha.gov/healthcare/infectious-diseases

[2] -World Health Organization (WHO). Implementation guide for vaccination of health workers. Geneva: WHO; 2022. Available from: https://www.who.int/publications/i/item/9789240052154

[3] Jenkin DC Mahgoub H Morales KF Lambach P Nguyen-Van-Tam JS. A rapid evidence appraisal of influenza vaccination in health workers: An important policy in an area of imperfect evidence. Vaccine X. 2019;2:100036. 10.1016/j.jvacx.2019.100036 31384750 PMC6668237

[4] Paterson P Meurice F Stanberry LR Glismann S Rosenthal SL Larson HJ. Vaccine hesitancy and healthcare providers. Vaccine. 2016;34(52):6700-6. 10.1016/j.vaccine.2016.10.042 27810314

[5] Karafillakis E Dinca I Apfel F Cecconi S Wűrz A Takacs J Vaccine hesitancy among healthcare workers in Europe: A qualitative study. Vaccine. 2016;34(41):5013-20. 10.1016/j.vaccine.2016.08.029 27576074

[6] Maltezou HC Katerelos P Poufta S Pavli A Maragos A Theodoridou M. Attitudes toward mandatory occupational vaccinations and vaccination coverage against vaccine-preventable diseases of health care workers in primary health care centers. Am J Infect Control. 2013;41(1):66-70. 10.1016/j.ajic.2012.01.028 22709989

[7] Peterson CJ Lee B Nugent K. COVID-19 vaccination hesitancy among healthcare workers-a review. Vaccines (Basel). 2022;10(6):948. 10.3390/vaccines10060948 35746556 PMC9227837

[8] LaMori J Feng X Pericone CD Mesa-Frias M Sogbetun O Kulczycki A. Hepatitis vaccination adherence and completion rates and factors associated with low compliance: A claims-based analysis of U.S. adults. PLoS One. 2022;17(2):e0264062. 10.1371/journal.pone.0264062 35176102 PMC8853527

[9] Pecoraro N Malatesta F Carpinelli L Fornino D Giordano C Moccia G Individual and contextual determinants of flu vaccination adherence: a university nudge intervention. Int J Environ Res Public Health. 2023;20(10):5900. 10.3390/ijerph20105900 37239626 PMC10218180

[10] European Centre for Disease Prevention and Control (ECDC). Seasonal influenza vaccination recommendations and coverage rates in EU/EEA Member States – An overview of vaccination recommendations for 2021–2022 and coverage rates for the 2018-2019 to 2020–21 influenza seasons. Stockholm: ECDC; 2023. Available from: https://www.ecdc.europa.eu/en/publications-data/seasonal-influenza-vaccination-recommendations-and-coverage-rates-eueea-member

[11] Verger P Botelho-Nevers E Garrison A Gagnon D Gagneur A Gagneux-Brunon A Vaccine hesitancy in health-care providers in Western countries: a narrative review. Expert Rev Vaccines. 2022;21(7):909-27. 10.1080/14760584.2022.2056026 35315308

[12] European Centre for Disease Prevention and Control (ECDC). Prevention of hepatitis B and C in the EU/EEA. Stockholm: ECDC; 2022. Available from: https://www.ecdc.europa.eu/en/publications-data/prevention-hepatitis-b-and-c-eueea

[13] Sandora TJ Gidengil CA Lee GM. Pertussis vaccination for health care workers. Clin Microbiol Rev. 2008;21(3):426-34. 10.1128/CMR.00003-08 18625679 PMC2493083

[14] Dubé È Ward JK Verger P MacDonald NE. Vaccine hesitancy, acceptance, and anti-vaccination: trends and future prospects for public health. Annu Rev Public Health. 2021;42(1):175-91. 10.1146/annurev-publhealth-090419-102240 33798403

[15] Pavlovic D Sahoo P Larson HJ Karafillakis E. Factors influencing healthcare professionals’ confidence in vaccination in Europe: a literature review. Hum Vaccin Immunother. 2022;18(1):2041360. 10.1080/21645515.2022.2041360 35290160 PMC9009961

[16] Oguz MM. Improving influenza vaccination uptake among healthcare workers by on-site influenza vaccination campaign in a tertiary children hospital. Hum Vaccin Immunother. 2019;15(5):1060-5. 10.1080/21645515.2019.1575164 30735439 PMC6605858

[17] Sartor C Tissot-Dupont H Zandotti C Martin F Roques P Drancourt M. Use of a mobile cart influenza program for vaccination of hospital employees. Infect Control Hosp Epidemiol. 2004;25(11):918-22. 10.1086/502320 15566024

[18] Boey L Roelants M Vandermeulen C. Increased vaccine uptake and less perceived barriers toward vaccination in long-term care facilities that use multi-intervention manual for influenza campaigns. Hum Vaccin Immunother. 2021;17(3):673-80. 10.1080/21645515.2020.1788327 32692943 PMC7993224

[19] Jarrett C Wilson R O’Leary M Eckersberger E Larson HJ SAGE Working Group on Vaccine Hesitancy. Strategies for addressing vaccine hesitancy - A systematic review. Vaccine. 2015;33(34):4180-90. 10.1016/j.vaccine.2015.04.040 25896377

[20] Lam PP Chambers LW MacDougall DM McCarthy AE. Seasonal influenza vaccination campaigns for health care personnel: systematic review. CMAJ. 2010;182(12):E542-8. 10.1503/cmaj.091304 20643836 PMC2934816

[21] Hollmeyer H Hayden F Mounts A Buchholz U. Review: interventions to increase influenza vaccination among healthcare workers in hospitals. Influenza Other Respir Viruses. 2013;7(4):604-21. 10.1111/irv.12002 22984794 PMC5781006

[22] Lytras T Kopsachilis F Mouratidou E Papamichail D Bonovas S. Interventions to increase seasonal influenza vaccine coverage in healthcare workers: A systematic review and meta-regression analysis. Hum Vaccin Immunother. 2016;12(3):671-81. 10.1080/21645515.2015.1106656 26619125 PMC4964628

[23] Rashid H Yin JK Ward K King C Seale H Booy R. Assessing interventions to improve influenza vaccine uptake among health care workers. Health Aff (Millwood). 2016;35(2):284-92. 10.1377/hlthaff.2015.1087 26858382

[24] Randi BA Sejas ONE Miyaji KT Infante V Lara AN Ibrahim KY A systematic review of adult tetanus-diphtheria-acellular (Tdap) coverage among healthcare workers. Vaccine. 2019;37(8):1030-7. 10.1016/j.vaccine.2018.12.046 30630694

[25] National Institute for Health and Care Excellence (NICE). Evidence review for multicomponent interventions to increase the uptake of routine vaccines. Vaccine uptake in the general population. Evidence review H. NICE Guideline, No. 218. London: NICE 2022. ISBN-13: 978-1-4731-4587-0. Available from: https://www.ncbi.nlm.nih.gov/books/NBK581891 35830534

[26] Page MJ McKenzie JE Bossuyt PM Boutron I Hoffmann TC Mulrow CD The PRISMA 2020 statement: an updated guideline for reporting systematic reviews. BMJ. 2021;372:n71. 10.1136/bmj.n71 33782057 PMC8005924

[27] Moral-Muñoz JA Herrera-Viedma E Santisteban-Espejo A Cobo MJ. Software tools for conducting bibliometric analysis in science: An up-to-date review. Prof Inf. 2020;29(1):29. 10.3145/epi.2020.ene.03

[28] World Health Organization (WHO). Classifying health workers. Mapping occupations to the international standard classification. Geneva: WHO; 2019. Available from: https://www.who.int/publications/m/item/classifying-health-workers

[29] International Standard Classification of Occupations (ISCO). ISCO-08 Structure, index correspondence with ISCO-88. Geneva: International Labour Organization; 2016. Available from: https://www.ilo.org/public/english/bureau/stat/isco/isco08

[30] World Health Organization (WHO). Vaccination in acute humanitarian emergencies: a framework for decision making, for details on cholera vaccination in response to outbreaks. Geneva: WHO. [Accessed: 2 Dec 2022]. Available from: https://www.who.int/publications/i/item/WHO-IVB-17.03

[31] The EndNote Team. EndNote. Version 20. Philadelphia: Clarivate; 2013. Available from: https://endnote.com/product-details?language=en

[32] Ouzzani M Hammady H Fedorowicz Z Elmagarmid A. Rayyan-a web and mobile app for systematic reviews. Syst Rev. 2016;5(1):210. 10.1186/s13643-016-0384-4 27919275 PMC5139140

[33] National Institutes of Health (NIH). Quality assessment of controlled intervention studies. Bethesda: NIH. [Accessed: 23 Feb 2023]. Available from: https://www.nhlbi.nih.gov/health-pro/guidelines/in-develop/cardiovascular-risk-reduction/tools/cohort

[34] Sterne JAC Savović J Page MJ Elbers RG Blencowe NS Boutron I RoB 2: a revised tool for assessing risk of bias in randomised trials. BMJ. 2019;366:l4898. 10.1136/bmj.l4898 31462531

[35] Sterne JA Hernán MA Reeves BC Savović J Berkman ND Viswanathan M ROBINS-I: a tool for assessing risk of bias in non-randomised studies of interventions. BMJ. 2016;355:i4919. 10.1136/bmj.i4919 27733354 PMC5062054

[36] DerSimonian R Laird N. Meta-analysis in clinical trials. Control Clin Trials. 1986;7(3):177-88. 10.1016/0197-2456(86)90046-2 3802833

[37] Clopper CJ Pearson ES. The use of confidence or fiducial limits illustrated in the case of the binomial. Biometrika. 1934;26(4):404-13. 10.1093/biomet/26.4.404

[38] Higgins JPT Thompson SG Deeks JJ Altman DG. Measuring inconsistency in meta-analyses. BMJ. 2003;327(7414):557-60. 10.1136/bmj.327.7414.557 12958120 PMC192859

[39] Viechtbauer W Cheung MW-L. Outlier and influence diagnostics for meta-analysis. Res Synth Methods. 2010;1(2):112-25. 10.1002/jrsm.11 26061377

[40] Egger M Davey Smith G Schneider M Minder C. Bias in meta-analysis detected by a simple, graphical test. BMJ. 1997;315(7109):629-34. 10.1136/bmj.315.7109.629 9310563 PMC2127453

[41] R Core Team. R: A language and environment for statistical computing. Vienna: R Foundation for Statistical Computing; 2021. Available from: https://www.R-project.org

[42] Babcock HM Gemeinhart N Jones M Dunagan WC Woeltje KF. Mandatory influenza vaccination of health care workers: translating policy to practice. Clin Infect Dis. 2010;50(4):459-64. 10.1086/650752 20064039

[43] Gołębiak I Okręglicka K Kanecki K Nitsch-Osuch A. The impact of selected educational and information interventions on the coverage rate and attitudes to influenza vaccination in nursing staff. Med Pr. 2020;71(6):665-85. 33024339 10.13075/mp.5893.00980

[44] Qureshi AM Hughes NJM Murphy E Primrose WR. Factors influencing uptake of influenza vaccination among hospital-based health care workers. Occup Med (Lond). 2004;54(3):197-201. 10.1093/occmed/kqg087 15133144

[45] Schmidtke KA Nightingale PG Reeves K Gallier S Vlaev I Watson SI Randomised controlled trial of a theory-based intervention to prompt front-line staff to take up the seasonal influenza vaccine. BMJ Qual Saf. 2020;29(3):189-97. 10.1136/bmjqs-2019-009775 31383723 PMC7061920

[46] Thomas DR Winsted B Koontz C. Improving neglected influenza vaccination among healthcare workers in long-term care. J Am Geriatr Soc. 1993;41(9):928-30. 10.1111/j.1532-5415.1993.tb06757.x 8409179

[47] Abramson ZH Avni O Levi O Miskin IN. Randomized trial of a program to increase staff influenza vaccination in primary care clinics. Ann Fam Med. 2010;8(4):293-8. 10.1370/afm.1132 20644183 PMC2906523

[48] Bert F Thomas R Lo Moro G Scarmozzino A Silvestre C Zotti CM A new strategy to promote flu vaccination among health care workers: Molinette Hospital’s experience. J Eval Clin Pract. 2020;26(4):1205-11. 10.1111/jep.13295 31697012

[49] Borgey F Henry L Lebeltel J Lescure P Le Coutour X Vabret A Effectiveness of an intervention campaign on influenza vaccination of professionals in nursing homes: A cluster-randomized controlled trial. Vaccine. 2019;37(10):1260-5. 10.1016/j.vaccine.2019.01.066 30738645

[50] Butteri MJ Radu C Huq F Wiglesworth A Durso SC Bellantoni M. Flu in 15: a novel 15-minute education program to promote acceptance of the influenza vaccine among health care workers. J Am Med Dir Assoc. 2010;11(7):523-7. 10.1016/j.jamda.2010.04.001 20816342

[51] Calderon M Feja KN Ford P Frenkel LD Gram A Spector D Implementation of a pertussis immunization program in a teaching hospital: an argument for federally mandated pertussis vaccination of health care workers. Am J Infect Control. 2008;36(6):392-8. 10.1016/j.ajic.2007.10.027 18675144

[52] Chambers LW Crowe L Lam PP MacDougall D McNeil S Roth V A new approach to improving healthcare personnel influenza immunization programs: a randomized controlled trial. PLoS One. 2015;10(3):e0118368. 10.1371/journal.pone.0118368 25781888 PMC4363667

[53] Chittaro M Turello D Calligaris L Farneti F Faruzzo A Fiappo E Impact of vaccinating HCWs on the ward and possible influence of avian flu threat. Infection. 2009;37(1):29-33. 10.1007/s15010-008-8002-6 19139813

[54] Cozza V Alfonsi V Rota MC Paolini V Ciofi degli Atti ML. Promotion of influenza vaccination among health care workers: findings from a tertiary care children’s hospital in Italy. BMC Public Health. 2015;15(1):697. 10.1186/s12889-015-2067-9 26204896 PMC4513703

[55] Currat M Lazor-Blanchet C Zanetti G. Promotion of the influenza vaccination to hospital staff during pre-employment health check: a prospective, randomised, controlled trial. J Occup Med Toxicol. 2020;15(1):34. 10.1186/s12995-020-00285-w 33292400 PMC7672907

[56] Dey P Halder S Collins S Benons L Woodman C. Promoting uptake of influenza vaccination among health care workers: a randomized controlled trial. J Public Health Med. 2001;23(4):346-8. 10.1093/pubmed/23.4.346 11873900

[57] de Juanes JR García de Codes A Arrazola MP Jaén F Sanz MI González A. Influenza vaccination coverage among hospital personnel over three consecutive vaccination campaigns (2001-2002 to 2003-2004). Vaccine. 2007;25(1):201-4. 10.1016/j.vaccine.2005.10.057 17011084

[58] Frenzel E Chemaly RF Ariza-Heredia E Jiang Y Shah DP Thomas G Association of increased influenza vaccination in health care workers with a reduction in nosocomial influenza infections in cancer patients. Am J Infect Control. 2016;44(9):1016-21. 10.1016/j.ajic.2016.03.024 27158088

[59] Frisina PG Ingraffia ST Brown TR Munene EN Pletcher JR Kolligian J. Increasing influenza immunization rates among healthcare providers in an ambulatory-based, University Healthcare Setting. Int J Qual Health Care. 2019;31(9):698-703. 10.1093/intqhc/mzy247 30624657

[60] Gilardi F Castelli Gattinara G Vinci MR Ciofi Degli Atti M Santilli V Brugaletta R Seasonal influenza vaccination in health care workers. A pre-post intervention study in an Italian paediatric hospital. Int J Environ Res Public Health. 2018;15(5):841. 10.3390/ijerph15050841 29695117 PMC5981880

[61] Heinrich-Morrison K McLellan S McGinnes U Carroll B Watson K Bass P An effective strategy for influenza vaccination of healthcare workers in Australia: experience at a large health service without a mandatory policy. BMC Infect Dis. 2015;15(1):42. 10.1186/s12879-015-0765-7 25656220 PMC4328539

[62] Jiang C Whitmore-Sisco L Gaur AH Adderson EE Tdap Working Group. A quality improvement initiative to increase Tdap (tetanus, diphtheria, acellular pertussis) vaccination coverage among direct health care providers at a children’s hospital. Vaccine. 2018;36(2):214-9. 10.1016/j.vaccine.2017.11.071 29217370

[63] Kim H Lindley MC Dube D Kalayil EJ Paiva KA Raymond P. Evaluation of the impact of the 2012 Rhode Island health care worker influenza vaccination regulations: implementation process and vaccination coverage. J Public Health Manag Pract. 2015;21(3):E1-9. 10.1097/PHH.0000000000000128 25105280 PMC4736136

[64] Kimura AC Nguyen CN Higa JI Hurwitz EL Vugia DJ. The effectiveness of vaccine day and educational interventions on influenza vaccine coverage among health care workers at long-term care facilities. Am J Public Health. 2007;97(4):684-90. 10.2105/AJPH.2005.082073 17329659 PMC1829357

[65] Kuntz JL Holley S Helms CM Cavanaugh JE Vande Berg J Herwaldt LA Use of a pandemic preparedness drill to increase rates of influenza vaccination among healthcare workers. Infect Control Hosp Epidemiol. 2008;29(2):111-5. 10.1086/526434 18179365

[66] Leitmeyer K Buchholz U Kramer M Schenkel K Stahlhut H Köllstadt M Influenza vaccination in German health care workers: effects and findings after two rounds of a nationwide awareness campaign. Vaccine. 2006;24(47-48):7003-8. 10.1016/j.vaccine.2006.04.040 16730866

[67] Looijmans-van den Akker I van Delden JJM Verheij TJM van der Sande MAB van Essen GA Riphagen-Dalhuisen J Effects of a multi-faceted program to increase influenza vaccine uptake among health care workers in nursing homes: A cluster randomised controlled trial. Vaccine. 2010;28(31):5086-92. 10.1016/j.vaccine.2010.05.003 20580740

[68] Marwaha S Lorv B Henseleit S Iroanyah N. GET POKED: Comparing an incentive-based flu campaign with vaccinate-or-mask policies to boost influenza vaccination rates among healthcare workers. Healthc Q. 2016;18(4):73-9. 10.12927/hcq.2016.24546 27009712

[69] Nace DA Handler SM Hoffman EL Perera S. Impact of the raising immunizations safely and effectively (RISE) program on healthcare worker influenza immunization rates in long term care settings. J Am Med Dir Assoc. 2012;13(9):806-10. 10.1016/j.jamda.2012.08.016 23031265 PMC3650646

[70] Paranthaman K McCarthy N Rew V van Zoelen S Cockerill L. Pertussis vaccination for healthcare workers: staff attitudes and perceptions associated with high coverage vaccination programmes in England. Public Health. 2016;137:196-9. 10.1016/j.puhe.2016.02.033 27026252

[71] Podczervinski S Stednick Z Helbert L Davies J Jagels B Gooley T Employee influenza vaccination in a large cancer center with high baseline compliance rates: comparison of carrot versus stick approaches. Am J Infect Control. 2015;43(3):228-33. 10.1016/j.ajic.2014.11.025 25728148 PMC4372134

[72] Rakita RM Hagar BA Crome P Lammert JK. Mandatory influenza vaccination of healthcare workers: a 5-year study. Infect Control Hosp Epidemiol. 2010;31(9):881-8. 10.1086/656210 20653445

[73] Ribner BS Hall C Steinberg JP Bornstein WA Chakkalakal R Emamifar A Use of a mandatory declination form in a program for influenza vaccination of healthcare workers. Infect Control Hosp Epidemiol. 2008;29(4):302-8. 10.1086/529586 18462141

[74] Rodríguez-Fernández R Martínez-López AB Pérez-Moreno J González-Sánchez MI González-Martínez F Hernández-Sampelayo T Impact of an influenza vaccine educational programme on healthcare personnel. Epidemiol Infect. 2016;144(11):2290-4. 10.1017/S0950268816000716 27053135 PMC9150534

[75] Rothan-Tondeur M Filali-Zegzouti Y Belmin J Lejeune B Golmard JL de Wazières B ORIG association. Assessment of healthcare worker influenza vaccination program in French geriatric wards: a cluster-randomized controlled trial. Aging Clin Exp Res. 2010;22(5-6):450-5. 10.1007/BF03337740 19966539

[76] Rothan-TondeurMFilali-ZegzoutiYGolmardJ-LDe WazieresBPietteFCarratF Randomised active programs on healthcare workers’ flu vaccination in geriatric health care settings in France: the VESTA study. J Nutr Health Aging. 2011;15(2):126-32. 10.1007/s12603-011-0025-521365166

[77] Saunier F Berthelot P Mottet-Auselo B Pelissier C Fontana L Botelho-Nevers E Impact of a decision-aid tool on influenza vaccine coverage among HCW in two French hospitals: A cluster-randomized trial. Vaccine. 2020;38(36):5759-63. 10.1016/j.vaccine.2020.07.011 32684500

[78] Smedley J Palmer C Baird J Barker M. A survey of the delivery and uptake of influenza vaccine among health care workers. Occup Med (Lond). 2002;52(5):271-6. 10.1093/occmed/52.5.271 12181376

[79] Smithers P Murray SB Stewart S Skull S. Hospital health care worker (HCW) vaccination coverage after implementation of an HCW vaccination policy. Aust Health Rev. 2003;26(1):76-83. 10.1071/AH030076 15485377

[80] Tapiainen T Bär G Schaad UB Heininger U. Influenza vaccination among healthcare workers in a university children’s hospital. Infect Control Hosp Epidemiol. 2005;26(11):855-8. 10.1086/502508 16320981

[81] Walther K Burckhardt MA Erb T Heininger U. Implementation of pertussis immunization in health-care personnel. Vaccine. 2015;33(17):2009-14. 10.1016/j.vaccine.2015.03.013 25776922

[82] Zimmerman RK Nowalk MP Lin CJ Raymund M Fox DE Harper JD Factorial design for improving influenza vaccination among employees of a large health system. Infect Control Hosp Epidemiol. 2009;30(7):691-7. 10.1086/598343 19489716

[83] Llupià A Mena G Olivé V Quesada S Aldea M Sequera VG Evaluating influenza vaccination campaigns beyond coverage: a before-after study among health care workers. Am J Infect Control. 2013;41(8):674-8. 10.1016/j.ajic.2013.04.006 23896285

[84] Conte A Quattrin R Filiputti E Cocconi R Arnoldo L Tricarico P Promotion of flu vaccination among healthcare workers in an Italian academic hospital: An experience with tailored web tools. Hum Vaccin Immunother. 2016;12(10):2628-33. 10.1080/21645515.2016.1186319 27245587 PMC5085013

[85] Ofstead CL Amelang MR Wetzler HP Tan L. Moving the needle on nursing staff influenza vaccination in long-term care: Results of an evidence-based intervention. Vaccine. 2017;35(18):2390-5. 10.1016/j.vaccine.2017.03.041 28351733

[86] Song JY Park CW Jeong HW Cheong HJ Kim WJ Kim SR. Effect of a hospital campaign for influenza vaccination of healthcare workers. Infect Control Hosp Epidemiol. 2006;27(6):612-7. 10.1086/504503 16755482

[87] Reñosa MDC Landicho J Wachinger J Dalglish SL Bärnighausen K Bärnighausen T Nudging toward vaccination: a systematic review. BMJ Glob Health. 2021;6(9):e006237. 10.1136/bmjgh-2021-006237 34593513 PMC8487203

[88] Lipsitch M Jha A Simonsen L. Observational studies and the difficult quest for causality: lessons from vaccine effectiveness and impact studies. Int J Epidemiol. 2016;45(6):2060-74. 10.1093/ije/dyw124 27453361 PMC5841615

[89] Hollingsworth R El Guerche-Séblain C Tsai T Vasiliev Y Lee S Bright H Assessment of the benefits of seasonal influenza vaccination: Elements of a framework to interpret estimates of vaccine effectiveness and support robust decision-making and communication. Influenza Other Respir Viruses. 2021;15(1):164-74. 10.1111/irv.12786 32885610 PMC7767949

[90] Razzaghi H Srivastav A de Perio MA Laney AS Black CL. Influenza and COVID-19 Vaccination Coverage Among Health Care Personnel - United States, 2021-22. MMWR Morb Mortal Wkly Rep. 2022;71(42):1319-26. 10.15585/mmwr.mm7142a2 36264832 PMC9590294

[91] Albanesi B Clari M Gonella S Chiarini D Aimasso C Mansour I The impact of COVID-19 on hospital-based workers influenza vaccination uptake: A two-year retrospective cohort study. J Occup Health. 2022;64(1):e12376. 10.1002/1348-9585.12376 36514845 PMC9748491

[92] Seale H Leask J Macintyre CR. Awareness, attitudes and behavior of hospital healthcare workers towards a mandatory vaccination directive: two years on. Vaccine. 2011;29(21):3734-7. 10.1016/j.vaccine.2011.03.050 21458607

[93] Squeri R Di Pietro A La Fauci V Genovese C. Healthcare workers’ vaccination at European and Italian level: a narrative review. Acta Biomed. 2019;90(9-S):45-53. 31517889 10.23750/abm.v90i9-S.8703 PMC7233663

[94] Butler R MacDonald NE SAGE Working Group on Vaccine Hesitancy. Diagnosing the determinants of vaccine hesitancy in specific subgroups: The Guide to Tailoring Immunization Programmes (TIP). Vaccine. 2015;33(34):4176-9. 10.1016/j.vaccine.2015.04.038 25896376

[95] Dini G Toletone A Sticchi L Orsi A Bragazzi NL Durando P. Influenza vaccination in healthcare workers: A comprehensive critical appraisal of the literature. Hum Vaccin Immunother. 2018;14(3):772-89. 10.1080/21645515.2017.1348442 28787234 PMC5861785

[96] Pelullo CP Della Polla G Napolitano F Di Giuseppe G Angelillo IF. Healthcare workers’ knowledge, attitudes, and practices about vaccinations: A cross-sectional study in Italy. Vaccines (Basel). 2020;8(2):148. 10.3390/vaccines8020148 32225018 PMC7348811

[97] MacDonald NE Butler R Dubé E. Addressing barriers to vaccine acceptance: an overview. Hum Vaccin Immunother. 2018;14(1):218-24. 10.1080/21645515.2017.1394533 29048975 PMC5791591

[98] MacDonald NE SAGE Working Group on Vaccine Hesitancy. Vaccine hesitancy: Definition, scope and determinants. Vaccine. 2015;33(34):4161-4. 10.1016/j.vaccine.2015.04.036 25896383

[99] Peters MDJ. Addressing vaccine hesitancy and resistance for COVID-19 vaccines. Int J Nurs Stud. 2022;131:104241. 10.1016/j.ijnurstu.2022.104241 35489108 PMC8972969

[100] Salgado CD Giannetta ET Hayden FG Farr BM. Preventing nosocomial influenza by improving the vaccine acceptance rate of clinicians. Infect Control Hosp Epidemiol. 2004;25(11):923-8. 10.1086/502321 15566025

[101] Gur-Arie R Jamrozik E Kingori P. No jab, no job? Ethical issues in mandatory COVID-19 vaccination of healthcare personnel. BMJ Glob Health. 2021;6(2):e004877. 10.1136/bmjgh-2020-004877 33597280 PMC7893205

[102] Galanakis E Jansen A Lopalco PL Giesecke J. Ethics of mandatory vaccination for healthcare workers. Euro Surveill. 2013;18(45):20627. 10.2807/1560-7917.ES2013.18.45.20627 24229791

[103] Limbu YB Gautam RK Pham L. The health belief model applied to COVID-19 vaccine hesitancy: a systematic review. Vaccines (Basel). 2022;10(6):973. 10.3390/vaccines10060973 35746581 PMC9227551

[104] Betsch C Schmid P Heinemeier D Korn L Holtmann C Böhm R. Beyond confidence: Development of a measure assessing the 5C psychological antecedents of vaccination. PLoS One. 2018;13(12):e0208601. 10.1371/journal.pone.0208601 30532274 PMC6285469

[105] European Centre for Disease Prevention and Control (ECDC). Facilitating COVID-19 vaccination acceptance and uptake in the EU/EEA. Stockholm: ECDC; 2021. Available from: https://www.ecdc.europa.eu/en/publications-data/facilitating-covid-19-vaccination-acceptance-and-uptake

[106] Geiger M Rees F Lilleholt L Santana AP Zettler I Wilhelm O Measuring the 7Cs of vaccination readiness. Eur J Psychol Assess. 2021.

[107] Rees F Geiger M Lilleholt L Zettler I Betsch C Böhm R Measuring parents’ readiness to vaccinate themselves and their children against COVID-19. Vaccine. 2022;40(28):3825-34. 10.1016/j.vaccine.2022.04.091 35623906 PMC9069251

[108] Verger P Fressard L Soveri A Dauby N Fasce A Karlsson L An instrument to measure psychosocial determinants of health care professionals’ vaccination behavior: Validation of the Pro-VC-Be questionnaire. Expert Rev Vaccines. 2022;21(5):693-709. 10.1080/14760584.2022.2046467 35238274

[109] Jama A Appelqvist E Kulane A Karregård S Rubin J Nejat S Design and implementation of tailored intervention to increase vaccine acceptance in a Somali community in Stockholm, Sweden - based on the Tailoring Immunization Programmes approach. Public Health Pract (Oxf). 2022;4:100305. 10.1016/j.puhip.2022.100305 36570400 PMC9773050

[110] World Health Organization Regional Office for Europe (WHO/Europe). TIP: Tailoring Immunization Programmes. Copenhagen: WHO/Europe; 2019. Available from: https://www.who.int/europe/publications/i/item/9789289054492

[111] Elliott TR Perrin PB Powers MB Jacobi KS Warren AM. Predictors of vaccine hesitancy among health care workers during the COVID-19 pandemic. Int J Environ Res Public Health. 2022;19(12):7123. 10.3390/ijerph19127123 35742372 PMC9222587

[112] La Torre G Mannocci A Ursillo P Bontempi C Firenze A Panico MG Prevalence of influenza vaccination among nurses and ancillary workers in Italy: systematic review and meta analysis. Hum Vaccin. 2011;7(7):728-33. 10.4161/hv.7.7.15413 21705859

[113] Wilson R Zaytseva A Bocquier A Nokri A Fressard L Chamboredon P Vaccine hesitancy and self-vaccination behaviors among nurses in southeastern France. Vaccine. 2020;38(5):1144-51. 10.1016/j.vaccine.2019.11.018 31810781

[114] Metelli S Chaimani A. Challenges in meta-analyses with observational studies. Evid Based Ment Health. 2020;23(2):83-7. 10.1136/ebmental-2019-300129 32139442 PMC10231593

[115] Concato J Shah N Horwitz RI. Randomized, controlled trials, observational studies, and the hierarchy of research designs. N Engl J Med. 2000;342(25):1887-92. 10.1056/NEJM200006223422507 10861325 PMC1557642

[116] Thomas RE Jefferson T Lasserson TJ. Influenza vaccination for healthcare workers who care for people aged 60 or older living in long-term care institutions. Cochrane Database Syst Rev. 2016;2016(6):CD005187. 10.1002/14651858.CD005187.pub5 27251461 PMC8504984

[117] Borenstein M Hedges LV Higgins JPT Rothstein HR. A basic introduction to fixed-effect and random-effects models for meta-analysis. Res Synth Methods. 2010;1(2):97-111. 10.1002/jrsm.12 26061376

[118] Turner RM Bird SM Higgins JPT. The impact of study size on meta-analyses: examination of underpowered studies in Cochrane reviews. PLoS One. 2013;8(3):e59202. 10.1371/journal.pone.0059202 23544056 PMC3609745

