# Supplementary material for: Effectiveness of interventions to increase healthcare workers’ adherence to vaccination against vaccine-preventable diseases: a systematic review and meta-analysis, 1993 to 2022
Source: Euro Surveill. 2024 Feb 29;29(9):2300276. doi: 10.2807/1560-7917.ES.2024.29.9.2300276 (PMC10986662; doi:10.2807/1560-7917.ES.2024.29.9.2300276)

This supplementary material is hosted by Eurosurveillance as supporting information alongside the article “Effectiveness of interventions to increase healthcare workers’ adherence to vaccination against vaccine-preventable diseases. A systematic review and meta-analysis”, on behalf of the authors, who remain responsible for the accuracy and appropriateness of the content. The same standards for ethics, copyright, attributions and permissions as for the article apply. Supplements are not edited by Eurosurveillance and the journal is not responsible for the maintenance of any links or email addresses provided therein.

Supplementary Table S1. PRISMA Checklist 2020

Section and Topic	Item #	Checklist item	Location where item is reported
**TITLE**			
Title	1	Identify the report as a systematic review.	Page 1
**ABSTRACT**			
Abstract	2	See the PRISMA 2020 for Abstracts checklist.	Page 1
**INTRODUCTION**			
Rationale	3	Describe the rationale for the review in the context of existing knowledge.	Page 2-3
Objectives	4	Provide an explicit statement of the objective(s) or question(s) the review addresses.	Page 4
**METHODS**			
Eligibility criteria	5	Specify the inclusion and exclusion criteria for the review and how studies were grouped for the syntheses.	Page 5
Information sources	6	Specify all databases, registers, websites, organisations, reference lists and other sources searched or consulted to identify studies. Specify the date when each source was last searched or consulted.	Pages 4-5
Search strategy	7	Present the full search strategies for all databases, registers and websites, including any filters and limits used.	Page 4 Supplementary Table 2
Selection process	8	Specify the methods used to decide whether a study met the inclusion criteria of the review, including how many reviewers screened each record and each report retrieved, whether they worked independently, and if applicable, details of automation tools used in the process.	Page 6
Data collection process	9	Specify the methods used to collect data from reports, including how many reviewers collected data from each report, whether they worked independently, any processes for obtaining or confirming data from study investigators, and if applicable, details of automation tools used in the process.	Page 6
Data items	10a	List and define all outcomes for which data were sought. Specify whether all results that were compatible with each outcome domain in each study were sought (e.g. for all measures, time points, analyses), and if not, the methods used to decide which results to collect.	Page 5
	10b	List and define all other variables for which data were sought (e.g. participant and intervention characteristics, funding sources). Describe any assumptions made about any missing or unclear information.	Page 5-6
Study risk of bias assessment	11	Specify the methods used to assess risk of bias in the included studies, including details of the tool(s) used, how many reviewers assessed each study and whether they worked independently, and if applicable, details of automation tools used in the process.	Page 6
Effect measures	12	Specify for each outcome the effect measure(s) (e.g. risk ratio, mean difference) used in the synthesis or presentation of results.	Pages 6-7
Synthesis methods	13a	Describe the processes used to decide which studies were eligible for each synthesis (e.g. tabulating the study intervention characteristics and comparing against the planned groups for each synthesis (item #5)).	Pages 6-7
	13b	Describe any methods required to prepare the data for presentation or synthesis, such as handling of missing summary statistics, or data conversions.	Pages 6-7
	13c	Describe any methods used to tabulate or visually display results of individual studies and syntheses.	Pages 6-7
	13d	Describe any methods used to synthesize results and provide a rationale for the choice(s). If meta-analysis was performed, describe the model(s), method(s) to identify the presence and extent of statistical heterogeneity, and software package(s) used.	Pages 6-7

Section and Topic	Item #	Checklist item	Location where item is reported
	13e	Describe any methods used to explore possible causes of heterogeneity among study results (e.g. subgroup analysis, meta-regression).	Pages 6-7
	13f	Describe any sensitivity analyses conducted to assess robustness of the synthesized results.	Pages 6-7
Reporting bias assessment	14	Describe any methods used to assess risk of bias due to missing results in a synthesis (arising from reporting biases).	Pages 6-7
Certainty assessment	15	Describe any methods used to assess certainty (or confidence) in the body of evidence for an outcome.	Pages 6-7
**RESULTS**			
Study selection	16a	Describe the results of the search and selection process, from the number of records identified in the search to the number of studies included in the review, ideally using a flow diagram.	Page 8
	16b	Cite studies that might appear to meet the inclusion criteria, but which were excluded, and explain why they were excluded.	Page 8
Study characteristics	17	Cite each included study and present its characteristics.	Pages 8-12
Risk of bias in studies	18	Present assessments of risk of bias for each included study.	Pages 12-13
Results of individual studies	19	For all outcomes, present, for each study: (a) summary statistics for each group (where appropriate) and (b) an effect estimate and its precision (e.g. confidence/credible interval), ideally using structured tables or plots.	Pages 13-15
Results of syntheses	20a	For each synthesis, briefly summarise the characteristics and risk of bias among contributing studies.	Pages 13-15
	20b	Present results of all statistical syntheses conducted. If meta-analysis was done, present for each the summary estimate and its precision (e.g. confidence/credible interval) and measures of statistical heterogeneity. If comparing groups, describe the direction of the effect.	Pages 13-15
	20c	Present results of all investigations of possible causes of heterogeneity among study results.	Pages 13-15
	20d	Present results of all sensitivity analyses conducted to assess the robustness of the synthesized results.	Pages 13-15
Reporting biases	21	Present assessments of risk of bias due to missing results (arising from reporting biases) for each synthesis assessed.	Pages 13-14
Certainty of evidence	22	Present assessments of certainty (or confidence) in the body of evidence for each outcome assessed.	Pages 13-14
**DISCUSSION**			
Discussion	23a	Provide a general interpretation of the results in the context of other evidence.	Pages 15-19
	23b	Discuss any limitations of the evidence included in the review.	Pages 19-20
	23c	Discuss any limitations of the review processes used.	Pages 19-20
	23d	Discuss implications of the results for practice, policy, and future research.	Page 20
**OTHER INFORMATION**			
Registration and protocol	24a	Provide registration information for the review, including register name and registration number, or state that the review was not registered.	Page 4
	24b	Indicate where the review protocol can be accessed, or state that a protocol was not prepared.	Page 4
	24c	Describe and explain any amendments to information provided at registration or in the protocol.	Page 4
Support	25	Describe sources of financial or non-financial support for the review, and the role of the funders or sponsors in the review.	Title Page or Page 20
Competing interests	26	Declare any competing interests of review authors.	Title Page or Page 20
Availability of data, code and other materials	27	Report which of the following are publicly available and where they can be found: template data collection forms; data extracted from included studies; data used for all analyses; analytic code; any other materials used in the review.	Supplementary Tables 2-7

From: Page MJ, McKenzie JE, Bossuyt PM, Boutron I, Hoffmann TC, Mulrow CD, et al. The PRISMA 2020 statement: an updated guideline for reporting systematic reviews. BMJ 2021;372:n71. doi: 10.1136/bmj.n71
For more information, visit: http://www.prisma-statement.org/

Supplementary Table S2. Search Syntaxs

Databases	Search Syntax	Results	Date of conduction
**PubMed**	((((((((((((("increas*"[Title/Abstract] OR "improv*"[Title/Abstract]) OR "impact"[Title/Abstract]) OR "educat*"[Title/Abstract]) OR "strateg*"[Title/Abstract]) OR "promot*"[Title/Abstract]) OR "encourag*"[Title/Abstract]) OR "intervention"[Title/Abstract]) OR "policy"[Title/Abstract]) OR "approach"[Title/Abstract]) OR "campaign"[Title/Abstract]) OR "program"[Title/Abstract]) AND (nurs*[Title/Abstract] OR ((Healthcare[Title/Abstract] OR health[Title/Abstract] OR hospital[Title/Abstract]) AND (worker*[Title/Abstract] OR professional*[Title/Abstract] OR personnel[Title/Abstract])) AND ((((((((((("Hesitancy"[Title/Abstract] OR "Reluctance"[Title/Abstract]) OR "resistance"[Title/Abstract]) OR "refusal"[Title/Abstract]) OR "acceptance"[Title/Abstract]) OR "willingness"[Title/Abstract]) OR "behavi*"[Title/Abstract]) OR "uptake"[Title/Abstract]) OR "rate*"[Title/Abstract]) OR "recei*"[Title/Abstract]) OR "aware*"[Title/Abstract]) OR "coverage"[Title/Abstract])) AND "vaccin*"[Title/Abstract]	6,911	1st January 2023
**CINHAL**	((MH "Personnel, Health Facility+") OR (MH "Nurses+") AND (hesitancy OR reluctance OR resistance OR refusal OR acceptance OR willingness OR behavi* OR uptake OR aware OR rate* OR recei* OR coverage) AND (MH "Immunization+") AND (increas* OR improv* OR impact* OR educat* OR strateg* OR promot* AND encourag* OR intervention OR policy OR improv* OR approach OR impact OR campaign* OR program)	254	1st January 2023
**Scopus**	(TITLE (vaccin*)) AND (TITLE-ABS (nurs* OR hospital*)) AND (TITLE (hesitancy OR reluctance OR resistance OR refusal OR acceptance OR willingness OR behavi* OR uptake OR rate* OR recei* OR aware OR coverage)) AND (TITLE (increas* OR improv* OR impact* OR educat* OR strateg* OR promot* AND encourag* OR intervention OR policy OR improv* OR approach OR impact OR campaign* OR program*))	220	1st January 2023
**Embase**	('vaccination coverage'/exp OR 'vaccination coverage' OR 'vaccination'/exp OR 'vaccination') AND ('nurse'/exp/mj OR 'nurse'/exp OR 'nurse') OR ('health care personnel'/exp/mj OR 'health care personnel') AND ('vaccine hesitancy'/exp OR 'vaccine hesitancy' OR 'health personnel attitude'/exp OR 'health personnel attitude' OR 'attitude to health'/exp OR 'attitude to health') AND ('management'/exp OR 'management') AND ([embase]/lim NOT ([embase]/lim AND [medline]/lim))	101	1st January 2023
**Web of Science**	TI=(vaccin*) AND TS=(nurs* OR hospital) OR TS=(healthcare) AND TS=(hesitancy OR reluctance OR resistance OR refusal OR acceptance OR willingness OR behavi* OR uptake OR rate* OR recei* OR aware OR coverage) AND TI=(increas* OR improv* OR impact* OR educat* OR strateg* OR promot* OR encourag* OR intervention OR policy OR improv* OR approach OR impact OR campaign* OR program*)	1,481	1st January 2023
**PsycInfo**	DE "Immunization" AND "Health Personnel Attitudes"	129	1st January 2023
**Cochrane Library**	#1 (vaccine OR vaccination)<br>#2 (hesitancy or reluctance or resistance or refusal or acceptance or willingness or behavi* or uptake or rate* or recei* or aware or coverage)<br>#3 (personnel or staff or nurs* or worker*)<br>#4 #1AND#2AND#3	1,415	1st January 2023
**Joanna Briggs Institute**	#1 ('Vaccination').mp. [mp=text, heading word, subject area node word, title]<br>#2 (hesitancy or reluctance or resistance or refusal or acceptance or willingness or behavi* or uptake or rate* or recei* or aware or coverage).mp. [mp=text, heading word, subject area node word, title]<br>#3 (increas* or improv* or impact or educat* or strateg* or promot* or encourag* or intervention or policy or improv* or approach or	57	1st January 2023

	impact or campaign or program).mp. [mp=text, heading word, subject area node word, title]<br>#4 (healthcare).mp. [mp=text, heading word, subject area node word, title]<br>#5 (nurs*).m_titl.<br>#6 4 OR 5<br>#7 1 AND 2 AND 3 AND 6		
**Other sources**	Registers<br>Prospero<br>ClinicalTrials.gov	50	1st January 2023

Supplementary Table S3. Exluded articles with reasons (n = 31)

First author, year	Reason for exclusion
Akerele, 2021	Not in line with the inclusion criteria – wrong population
Cantarelli, 2021	Not in line with the inclusion criteria – wrong population
Casalino, 2018	Not in line with the inclusion criteria – wrong outcome
Costantino, 2019	Not in line with the inclusion criteria – wrong study design
Donzel Godinot, 2021	Not in line with the inclusion criteria – wrong population
Doratotaj, 2008	Not in line with the inclusion criteria – wrong population
Gagneur, 2019	Not in line with the inclusion criteria – wrong outcome
Hayaward, 2006	Not in line with the inclusion criteria – wrong outcome
Hood, 2009	Not in line with the inclusion criteria – wrong population
McCullers, 2006	Not in line with the inclusion criteria – wrong population
McGrane, 2003	Not in line with the inclusion criteria – wrong outcome
MacDougall, 2016	Not in line with the inclusion criteria – wrong outcome
Munoz-Miralles, 2021	Not in line with the inclusion criteria – wrong study design
LaVela, 2015	Not in line with the inclusion criteria – wrong study design
Launay, 2014	Not in line with the inclusion criteria – wrong outcome
Lindley, 2006	Not in line with the inclusion criteria – wrong study design
Lopes, 2008	Not in line with the inclusion criteria – wrong study design
Looijmans-van den Akker, 2011	Not in line with the inclusion criteria – wrong study design
Lynch, 2015	Not in line with the inclusion criteria – wrong population
Polgreen, 2010	Not in line with the inclusion criteria – wrong study design
Paparone, 2015	Not in line with the inclusion criteria – wrong study design
Ribner, 2011	Not in line with the inclusion criteria – wrong study design
Riphagen-Dalhuisen, 2012	Not in line with the inclusion criteria – wrong study design
Riphagen-Dalhuisen, 2013	Not in line with the inclusion criteria – wrong outcome
Seale, 2011	Not in line with the inclusion criteria – wrong population
Stenqvist, 2006	Not in line with the inclusion criteria – wrong study design
Tarhan, 2006	Not in line with the inclusion criteria – wrong outcome
Thielmann, 2015	Untraceable from the source
Tognetto, 2020	Not in line with the inclusion criteria – wrong outcome
Venkat, 2012	Not in line with the inclusion criteria – wrong population
Wilde, 1999	Not in line with the inclusion criteria – wrong study design

Supplementary Table S4. Description of provided interventions' type, components and operational strategies (N = 48)

Article	Intervention type and component(s)	Operational strategies
		Contents (C)<br>Modalities of intervention delivering (M)<br>Used tools (T)<br>Frequency and Session (F);
Abramson<br>(Israel, 2010)	Multi-component educational + promotional + policy	*Educational strategy:*<br>Immunology, misconceptions on influenza vaccination (C);<br>Lecture session to the staff by GPs (M);<br>*Promotional strategy:*<br>Dissemination of vaccination guidelines<br>Posters and leaflets, email reminders to vaccination and in-services key figures informants (T);<br>*Policy strategy:*<br>Free in-wards vaccination;<br>Development of recommendation and promotional policy (M);<br>Dissemination of vaccination guidelines and data monitoring (T);
Babcock<br>(USA, 2010)	Multi-component educational + promotional + policy	*Educational strategy:*<br>Theoretical immunology and misconceptions on immunization (C);<br>Standardized educational materials (M);<br>Slideshows and fact-sheets (T);<br>*Promotional strategy:*<br>Influenza vaccinations (C);<br>Council meetings; Real-time vaccination tracking; Free in-services vaccination, multidisciplinary implementation team, "Town Hall Meetings" (M);<br>Internet site, letters meetings, in-house newspapers (T);<br>Bi-monthly meetings(F);<br>*Policy strategy:*<br>Mandatory and financial policy recommendations based on BJC occupational health services and exemptions (M);<br>Consent forms, real-time data evaluation; badge scanners (T);
Bert<br>(Italy, 2019)	Multi-component educational + promotional	*Educational strategy*<br>Direct educational sessions on perceptions and concerns related to vaccination (C);<br>Standardized educational and informative materials (M);<br>Slideshows, educational materials and posters (T);<br>*Promotional strategy:*<br>Knowledge on vaccination (C);<br>Free in-services moving vaccination; unit; (M);<br>Moving informative vaccination unit and delivering of materials (T);
Boey<br>(Belgium, 2021)	Multi-component educational + promotional	*Educational strategy:*<br>Theoretical immunology and virology (C);<br>Standard educational materials; role models, group sharing; (M);<br>Slideshows, educational materials and posters (T);<br>*Promotional strategy:*

		Knowledge on vaccination (C);<br>Personalized and certificate campaigns, manual redaction and multidisciplinary meetings (M);<br>Campaign kickoff event, electronic registration, free in-wards vaccinations, communication messages and channels, multiple and peer vaccinations (T);
Borgey<br>(France, 2019)	Multi-component educational + promotional + policy	Design of the intervention on WHO multimodal strategies<br>*Educational strategy:*<br>Influenza and vaccination (C);<br>Educational session on-site with the contribution of local leaders (M);<br>Short slideshow and videos(T);<br>2-minutes short videos (F);<br>*Promotional strategy:*<br>NS(C);<br>Reminders to vaccination, data monitoring and information diffusion, In-wards free immunization (M);<br>Posters, questionnaire and leaflets (T);<br>NS(C) and (F);<br>*Policy strategy:*<br>System change with free-in-facilities immunization (M);
Butteri<br>(USA, 2010)	Mono-component educational	*Educational strategy:*<br>Influenza and others' vaccinations types as Hepatitis B, contraindications and infectious transmission (C);<br>In-wards co-teaching small groups educations (M);<br>Slideshows, verbal presentation 1-page information handout, surveys (T);<br>15-minutes per week (F);
Calderon<br>(USA, 2008)	Multi-component educational + promotional	*Educational strategy:*<br>Theoretical immunology and virology (C);<br>Standard educational materials; role models, group sharing; (M);<br>Slideshows, educational materials and posters (T);<br>*Promotional strategy:*<br>Knowledge on vaccination (C);<br>Personalized and certificate campaigns, manual redaction and multidisciplinary meetings (M);<br>Campaign kickoff event, electronic registration, free in-wards vaccinations, communication messages and channels, multiple and peer vaccinations (T);
Chambers<br>(Canada, 2015)	Mono-component educational	*Educational strategy:*<br>Theoretical immunology, virology, infection prevention and best practice diffusion (C);<br>Theoretical lessons, plenary discussions, distance learning and internet forum discussion about vaccination opinions based on the Guide Tool Kit 5 methodology (M);<br>Active educational daily in-services learning (T);
Chittaro<br>(Italy, 2009)	Multi-component educational + promotional	*Educational strategy:*<br>Influenza vaccination (C);<br>Distribution of lectures and informative materials about influenza immunization from primary physician and nurse (M);<br>Slideshows and leaflets (T);<br>18-scheduled lectures (approximately 20<br>to 30 minutes) of 3-weeks (F);<br>*Promotional strategy:*

		Open in-wards free vaccination, data collection and monitoring from occupational health services(M);<br>Posters in common areas, leaflets and reminder letter (T);<br>1 week of free vaccination per 1 month per year (F);
Conte<br>(Italy, 2016)	Multi-component educational + promotional	*Educational strategy:*<br>Theoretical information about influenza and vaccination (C);<br>In-wards lessons (M);<br>Slideshows (T);<br>*Promotional strategy:*<br>Spots campaign, posters and leaflets distribution (M);<br>Intranet sharing of messages, information about vaccination. data collection and monitoring on vaccination; (T);
Cozza<br>(Italy, 2015)	Multi-component educational + promotional	*Educational strategy:*<br>Training about influenza and vaccination (C);<br>In-wards lessons (M);<br>Slideshows (T);<br>*Promotional strategy:*<br>Posters and leaflets distribution (M);<br>Information factsheets, in-paper forms and hospital sharing of information about immunizations;<br>Electronic immunization track and self-reported survey (T);
Currat<br>(Switzerland, 2020)	Mono-component educational	*Educational strategy:*<br>Theoretical, virology and infection prevention (C);<br>In-services learning and online lectures to pre employed HCWs (M);<br>Slideshows (T);<br>5-minutes of the theoretical lesson at the health check (F);
Dey<br>(UK, 2001)	Multi-component educational + promotional	*Educational strategy*<br>Theoretical, virology and misconceptions about vaccination (C);<br>Public health nurse in-services lecturers (M);<br>Briefings and slideshows (T);<br>*Promotional strategy*<br>Communicational and informative campaign (M);<br>Posters, leaflets in common areas and immunization monitoring(T);
de Juanes<br>(Spain, 2007)	Multi-component educational + promotional	*Educational strategy:*<br>Training education on influenza vaccination (C);<br>In-wards lessons (M);<br>Slideshows (T);<br>*Promotional strategy:*<br>In-wards passive immunization campaign and scheduling presentation (M);<br>Web informative bulletin, leaflets and slideshows (T);
Frenzel<br>(USA, 2016)	Multi-component educational + promotional + policy	*Educational strategy:*<br>Training education on influenza vaccination (C);<br>Informative and educational materials distribution (M);<br>Slideshows (T);<br>*Promotional strategy:*<br>Sign-in-sheets mobile carts, paper sign-in-sheets, posters, bulletin and meetings,

		Free in-wards vaccination, roaming service hours and on-site vaccinations clinics (T);<br>*Policy strategy:*<br>Mandatory influenza prevention program and guidelines implementation (M);
Frisina<br>(USA, 2019)	Multi-component educational + promotional	*Educational strategy:*<br>Training education on influenza vaccination (C);<br>Educational materials (M);<br>Slideshows and leaflets (T);<br>*Promotional strategy:*<br>CDC recommendations and influenza fact-sheets (C);<br>Informative materials distribution, fact-sheets and improvement teams (M);<br>Free-in wards vaccinations, monthly emails distribution and training; (T);
Gilardi<br>(Italy, 2018)	Multi-component educational + promotional	*Educational strategy:*<br>Immunology (C);<br>On-site lecturers and educational materials (M);<br>Slideshows and leaflets (T);<br>*Promotional strategy:*<br>CDC recommendations and influenza fact-sheets (C);<br>Fixed-stations, occupational medicine surveillance and visits (M);<br>On-site vaccinations, posters, letters, personal solicitations and leaflets, information factsheets, hospital website (T);
Golebiak<br>(Poland, 2020)	Mono-component educational	*Educational strategy*<br>Virus transmission channels, theoretical immunology, misconceptions on immunization (C);<br>Stationary training, distance learning and meeting with educators (M);<br>Slideshows, written materials and key-contents leaflets (T);<br>Meetings with educators after 2 weeks from lessons (F);
Heinrich – Morrison<br>(Australia, 2015)	Multi-component educational + promotional	*Educational strategy:*<br>Theoretical education on influenza (C);<br>Educational materials (M);<br>Slideshows and surveys on HCWs vaccination knowledge (T);<br>1 hour of lesson per week (F);<br>*Promotional strategy:*<br>CDC recommendations and influenza fact-sheets (C);<br>Broad vaccination campaigns and materials (M);<br>Leaflets, posters, web-based and in-services vaccination programs; weekly electronic and hospital meetings, social marketing campaign, coffee incentives to get vaccination (T);<br>3 hours of promotional meetings (F);
Jiang<br>(USA, 2018)	Multi-component educational + promotional	Development of intervention based on Model Understanding Success in Quality (MUSIQ)<br>*Educational strategy:*<br>Theoretical lectures on immunization and influenza (C);<br>In-vivo meeting and lessons (M);<br>Slideshows (T);<br>*Promotional strategy:*<br>Communicative-promotional campaign, multidisciplinary meetings (M);

		promotional materials, posters and web email targeting campaign, posters and leaflets, database monitoring and in-wards immunization, and routinely review of vaccination data(T);
Kim<br>(USA, 2018)	Multi-component educational + promotional	*Educational strategy:*<br>Theoretical education on influenza (C);<br>Theoretical lectures and digital materials on preparedness drill (M);<br>Slideshows (T);<br>*Promotional strategy:*<br>Communicative-promotional campaign (M);<br>Posters, leaflets incentives, immunization charts and diffusion of immunization strategies (T);
Kimura<br>(USA, 2007)	Multi-component educational + promotional	*Educational strategy*<br>Theoretical immunology and virology and misconceptions on immunization (C);<br>Theoretical lessons and videos (M);<br>Educational videos, Knowledge questionnaires distribution; 1-page of educational flyers (T);<br>10-minutes of educational videos (F);<br>*Promotional strategy*<br>Delivering of information material (M);<br>Poster distributions; informative vaccination days (T);
Kuntz<br>(USA, 2008)	Multi-component educational + promotional	*Educational strategy:*<br>Lessons on influenza vaccination (C);<br>Educational talks and sharing on influenza vaccination (M);<br>Web informative bulletin to HCWs with educational materials on influenza vaccination, educational talks, slideshows and multidisciplinary meetings, diffusion and sharing of published articles (T);<br>*Promotional strategy:*<br>Influenza transmission and importance of vaccination (C);<br>Communicative-promotional campaign;<br>Posters and web email targeting campaign (M);<br>Free-in wards nurses vaccinations on Hospital Emergency Incident Command System (HEICS) model, data monitoring and collection (T);<br>2-weeks (F);
Leitmeyer<br>(Germany, 2006)	Multi-component educational + promotional + policy	*Educational strategy:*<br>Theoretical education and training on influenza vaccination coverage (C);<br>In-house lessons (M);<br>Slideshows, materials sharing and online information packages; (T);<br>*Promotional strategy:*<br>Influenza transmission and importance of vaccination (C);<br>Promotional in-services campaign (M);<br>Posters, leaflets and evaluation surveys; mass mailing of informative and training materials and financial contribution to vaccination(T);<br>2 months (F);
LIupìa<br>(Spain, 2010)	Multi-component educational + promotional + policy	Developed on Professionals and Influenza Vaccination (PIVAC) framework;<br>*Educational strategy:*<br>Theoretical education and influenza transmission (C);<br>Educational materials (M);

		Website and blogs sharing on HCWs knowledge and experience of vaccination; (T);<br>*Promotional strategy:*<br>Promotional campaign in hospital and online (M);<br>Informative messages, promotional videos sharing, leaflets and brochures on influenza vaccination;<br>*Policy strategy:*<br>Financial contribution to vaccination; (T);
Looijmans-van den Akker<br>(The Netherlands, 2010)	Multi-component educational + promotional	Design on the strategy of Intervention Mapping<br>*Educational strategy*<br>Theoretical immunology and misconceptions on immunization (C);<br>Theoretical lessons (M);<br>Website creation, in-services groups discussions, slideshows, role-models videos; (T);<br>1-hour per 2 meetings of plenary presentation (F);<br>*Promotional strategy*<br>Vaccination types and importance (C);<br>Delivering of information material (M);<br>Outreach visits, HCWs personal invitation to get vaccination, posters, leaflets, messages personal invitations (T);
Marwaha<br>(Canada, 2016)	Multi-component educational + promotional	*Educational strategy:*<br>Theoretical education and influenza transmission; (C);<br>Educational materials (M);<br>Slideshows and surveys on HCWs vaccination knowledge (T);<br>*Promotional strategy:*<br>In-hospital promotional campaign on GETPOCKET strategy (M);<br>Informative messages, emails cards and promotional leaflets and informative materials on influenza vaccination (T);
Nace<br>(USA,2011)	Multi-component educational + promotional	Developed on the Raising Immunization Safety and Effectively (RISE) Program<br>*Educational strategy:*<br>Theoretical education and influenza transmission; (C);<br>Online education (M);<br>Website and blogs sharing on HCWs knowledge and experience of vaccination, diffuse training and communication program (T);<br>*Promotional strategy:*<br>In-hospital and online promotional campaign; (M);<br>Informative messages, promotional leaflets and informative materials on influenza vaccination (T);
Ofstead<br>(USA, 2017)	Multi-component educational + promotional	Developed on Health Belief Ecological Model<br>*Educational strategy:*<br>Theoretical education and influenza transmission based (C);<br>In-services education meetings (M);<br>Diffuse training and communication program, knowledge questionnaire development (T);<br>1 hour per week (F);<br>*Promotional strategy:*<br>Promotional campaign in hospital and online (M);<br>Leaflets and informative materials on influenza vaccination, electronic immunization roster tracking (T);
Oguz<br>(Turkey, 2019)	Mono-component policy	*Policy strategy:*<br>Influenza transmission and vaccination (C);

		Diffusion and sharing of promotional materials (M);<br>Free-in-wards immunization and mobile immunization team diffusion, recommendation distribution to HCWs, goal-setting worksheets, kick-off events, incentives, vaccination tracking roster, and facility-wide communication (T);
Paranthaman (UK, 2016)	Multi-component educational + promotional	*Educational strategy:*<br>Theoretical education on Tdap transmission; (C);<br>Educational materials (M);<br>Surveys on HCWs vaccination knowledge (T);<br>*Promotional strategy:*<br>In-hospital promotional campaign (M);<br>Informative messages and promotional leaflets on pertussis vaccination (T);
Podczervinski (USA, 2015)	Multi-component educational + promotional + policy	*Educational strategy:*<br>Theoretical education on vaccination and influenza (C);<br>In-services education meetings (M);<br>Diffuse training and one-to-one peer counseling and managers discussions on HCWs vaccination; (T);<br>*Promotional strategy:*<br>Diffusion of promotional materials and sharing (M);<br>Free-in-wards immunization and mobile immunization team diffusion, center-wide advertising and flu vaccination talks; Email reminders to get vaccination and weekly reminders to managers regarding non-compliant HCWs, center leadership support/involvement (T);<br>Promotional activities were provided from 6 to 8 weeks (F);<br>*Policy strategy:*<br>Incentives gift cards, non-mandatory penalty-based vaccination policy (T);
Qureshi (UK, 2004)	Multi-component educational + promotional	*Educational strategy:*<br>Theoretical education and influenza transmission (C);<br>Lectures on influenza and immunization (M);<br>Diffuse training and one-to-one peer counseling and managers discussions on HCWs vaccination; (T);<br>*Promotional strategy:*<br>In-hospital promotional campaign and informative distribution (M);<br>Leaflets and informative materials on influenza vaccination (T);
Rakita (USA, 2010)	Multi-component educational + promotional + policy	*Educational strategy:*<br>Theoretical education and influenza transmission (C);<br>Online and in-wards lectures on influenza and immunization, educational informative workshops and focus-group (M);<br>Diffuse training and one-to-one peer counseling and managers discussions on HCWs vaccination; (T);<br>*Promotional strategy:*<br>Multidisciplinary teams, in-wards and drive-in immunization campaign and vaccinations sites (M);<br>Leaflets and website informative materials on influenza vaccination, kick-off and incentives meeting to increase vaccination adherence among HCWs (T);<br>NS (C) and (F);<br>*Policy strategy:*<br>In-wards diffusion of CDC recommendation (M)<br>Diffuse training on CDC influenza recommendations, mandatory vaccination (T);
Ribner (USA, 2008)	Multi-component	*Educational strategy:*<br>Theoretical education and influenza transmission (C);

	educational + promotional + policy	Lectures on influenza and immunization (M);<br>Diffuse training and one-to-one peer counseling and managers discussions on HCWs vaccination; (T);<br>*Promotional strategy:*<br>In-hospital promotional campaign and informative distribution (M);<br>Leaflets and informative materials on influenza vaccination;<br>*Policy strategy:*<br>Use of mandatory declination form and guidelines (T);
Rodriguez-Fernandez (Spain, 2016)	Mono-component educational	*Educational strategy:*<br>Training educational program on influenza immunization and the associated risks factors (C);<br>Theoretical lessons, plenary discussions, and forum discussion about vaccination opinions, active educational daily in-services learning (M);<br>NS (C) and (F);
Rothan – Tondeur (France, 2010)	Mono-component educational	*Educational strategy*<br>Theory about vaccination and vaccines (C);<br>In –services theoretical lessons by GPs (M);<br>Slideshows, leaflets and guides on vaccination (T);<br>2-hours of lessons (F);
Rothan – Tondeur (France, 2011)	Mono-component educational	*Educational strategy*<br>Theory about vaccination and vaccines (C);<br>In –services theoretical lessons by GPs (M);<br>Slideshows, leaflets and guides on vaccination (T);<br>2-hours of lessons (F);
Sartor (France, 2004)	Multi-component educational + promotional	*Educational strategy:*<br>Theoretical education and influenza transmission (C);<br>Lectures on influenza and immunization (M);<br>Diffuse training and one-to-one peer counseling and managers discussions on HCWs vaccination; (T);<br>*Promotional strategy:*<br>In-hospital promotional campaign and informative distribution (M);<br>Leaflets and informative materials on influenza vaccination (T);
Saunier (France, 2020)	Mono-component informative	*Informative strategy*<br>Theory about vaccination and vaccines (C);<br>In-services distribution of information materials (M);<br>Leaflets as DA and informative materials based on the Ottawa Decision Aid Tool (OIDA) (T);
Schmidtke (UK, 2020)	Multi-component educational + promotional + policy	Designed on Nudge Theory<br>*Educational strategy:*<br>Theoretical immunology and misconceptions on immunization (C);<br>Theoretical lessons and groups discussions (M);<br>Slideshow, face-to-face teams briefings (T);<br>*Promotional strategy:*<br>Influenza vaccinations (C);<br>Social media campaign and information meetings (M);<br>Dissemination of leaflets and posters (T);<br>2 hours of informations meetings per week (F);

		Policy strategy:<br>Development and dissemination of recommendation policies and in-services free vaccination (M);<br>Emails and newsletters (T);
Smedley<br>(UK, 2002)	Multi-component educational + promotional	*Educational strategy:*<br>Theoretical education and influenza transmission (C);<br>Lectures on influenza and immunization (M);<br>Diffuse training and managers discussions on HCWs vaccination (T);<br>*Promotional strategy:*<br>In-hospital promotional campaign and informative distribution (M);<br>Leaflets and informative materials on influenza vaccination (T);
Smithers<br>(Australia, 2003)	Mono-component policy	*Policy strategy:*<br>Influenza transmission (C);<br>Recommendations development and sharing (M);<br>Diffusion of recommendations materials on immunization, leaflets and data collection on questionnaires data and immunization (T);
Song<br>(Korea, 2006)	Multi-component educational + promotional	*Educational strategy:*<br>Theoretical education and influenza transmission (C);<br>Lectures on influenza and immunization (M);<br>Diffuse training and discussions on HCWs vaccination, knowledge questionnaire distribution (T);<br>*Promotional strategy:*<br>In-hospital promotional campaign and informative distribution (M);<br>Leaflets and mobile carts system distribution on influenza vaccination; (T);
Tapiainen<br>(Switzerland, 2005)	Multi-component educational + promotional	*Educational strategy:*<br>Theoretical education and influenza transmission (C);<br>Lectures on influenza and immunization (M);<br>Diffuse training and discussions on HCWs vaccination (T);<br>*Promotional strategy:*<br>In-wards campaign and informative distribution (M);<br>In-wards campaign and informative distribution (T);
Thomas<br>(USA, 1993)	Multi-component educational + promotional + policy	*Educational strategy:*<br>Theoretical education and influenza transmission (C);<br>In-wards lectures on influenza and immunization, educational informative workshops and focus-group (M);<br>Diffuse training and discussions on HCWs vaccination (T);<br>*Promotional strategy:*<br>In-wards immunization campaign and vaccinations sites (M);<br>Leaflets, informative materials on influenza vaccination (T);<br>1 day per week until the end of intervention (F);<br>*Policy strategy:*<br>Diffuse of recommendations on influenza (M);<br>Leaflets and guidelines materials (T);<br>1-day in-service free immunization (F);
Walther<br>(Switzerland, 2015)	Multi-component	*Educational strategy:*<br>Theoretical education and influenza transmission (C);

	educational + promotional	Diffuse training on vaccination (M);<br>Lectures on influenza and immunization, educational informative workshops and focus-group (T);<br>*Promotional strategy:*<br>Promotional informative campaign (M);<br>Mandatory appointments on immunization, constant checking on immunization and vaccination status; (T);
Zimmerman (USA, 2009)	Multi-component educational + promotional	*Educational strategy:*<br>Theoretical education and influenza transmission (C);<br>Diffuse training on vaccination (M);<br>Lectures on influenza and immunization, educational informative workshops (T);<br>*Promotional strategy:*<br>Promotional activities and diffusion of immunization (M);<br>Free-in-wards vaccinations, in-wards campaign and informative distribution, leaflets and web informative bulletins distribution on influenza vaccination (T);

GPs: General Practitioners; KAB: Knowledge, Attitudes, Behaviors;

Supplementary Figure 5. Funnel plots

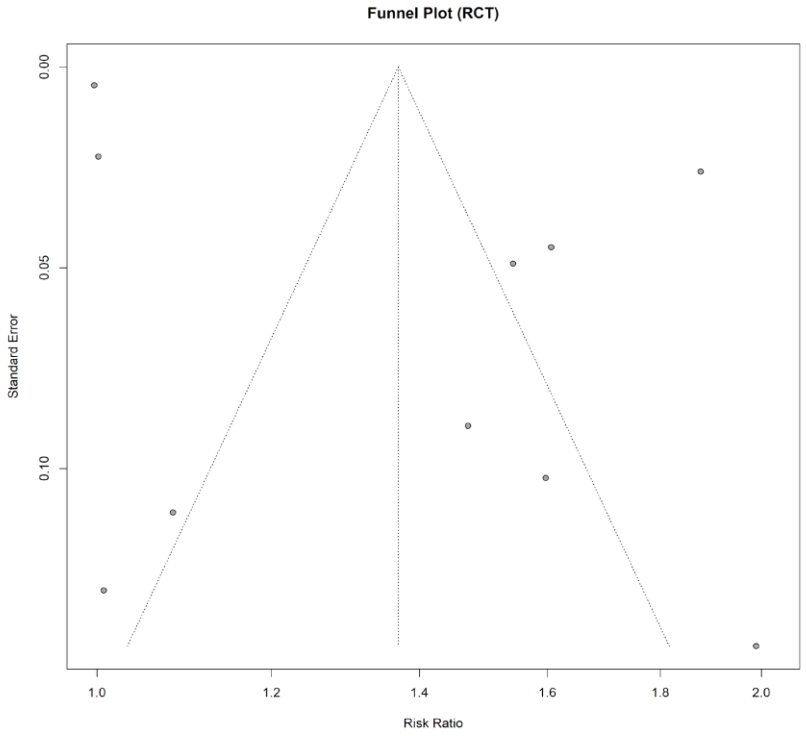

Funnel plots of RCT on seasonal influenza (n = 10)

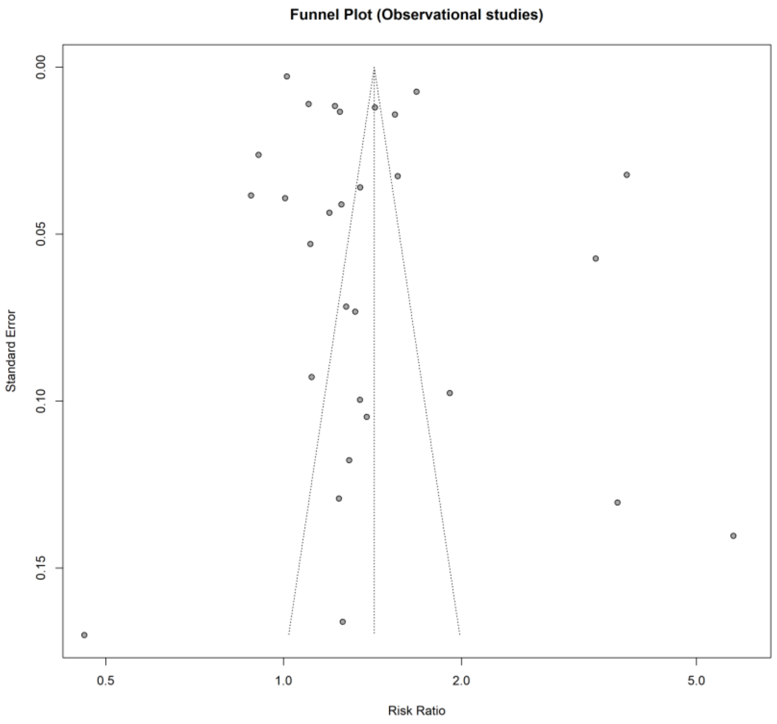

Funnel plots of observational studies on seasonal influenza (n = 29)

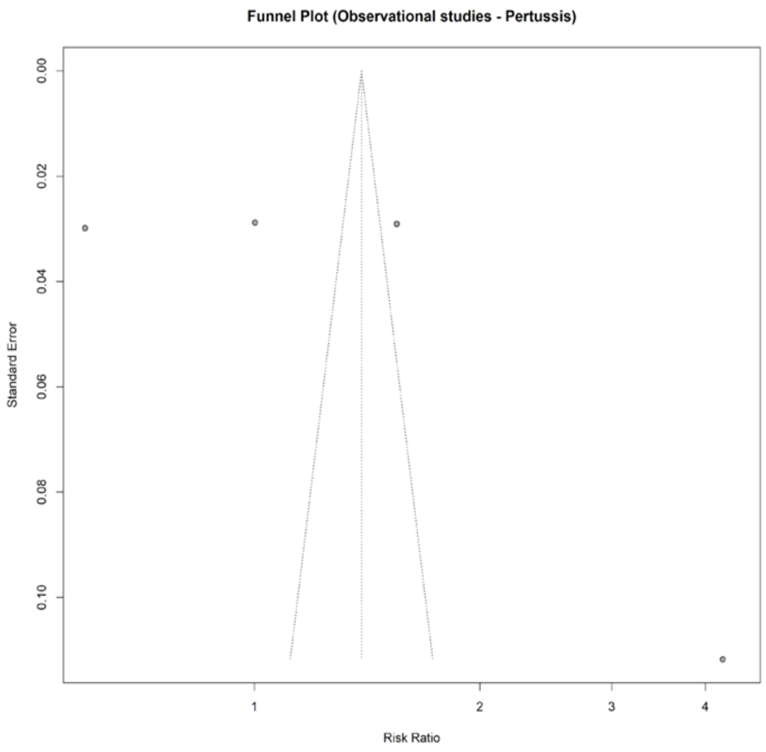

Funnel plots of observational studies on Tdap (n = 4)

Supplementary Figure 6. Leave-one-out sensitivity of RCT and observational studies for VPDs, figures 1 and 2 (n = 43)

(a) Leave-one-out sensitivity for seasonal influenza vaccination (RCT and observational studies)

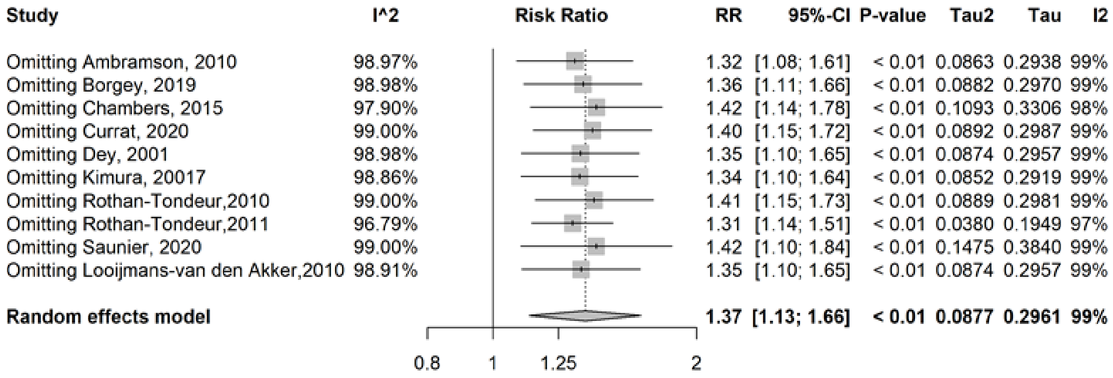

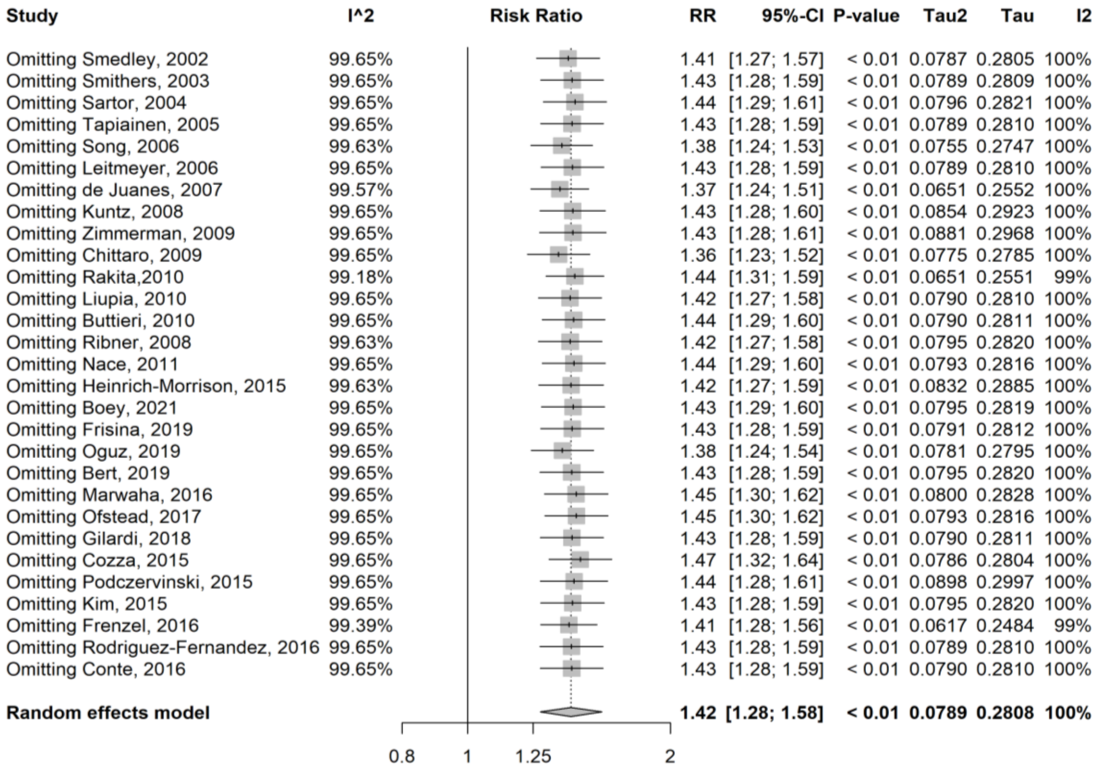

(b) Leave-one-out sensitivity for Tdap vaccination (observational studies)

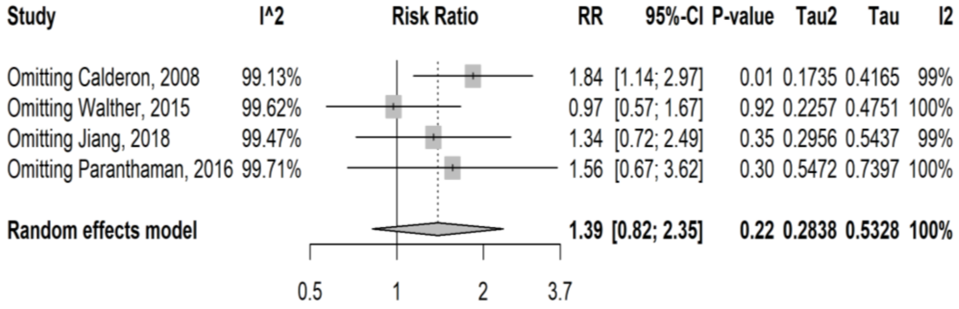

Supplementary Figure 7. Sensitivity analysis of RCT and observational studies for VPDs, figures 1 and 2 (n = 43)

(a) Sensitivity analysis for seasonal influenza vaccination (RCT and observational studies)

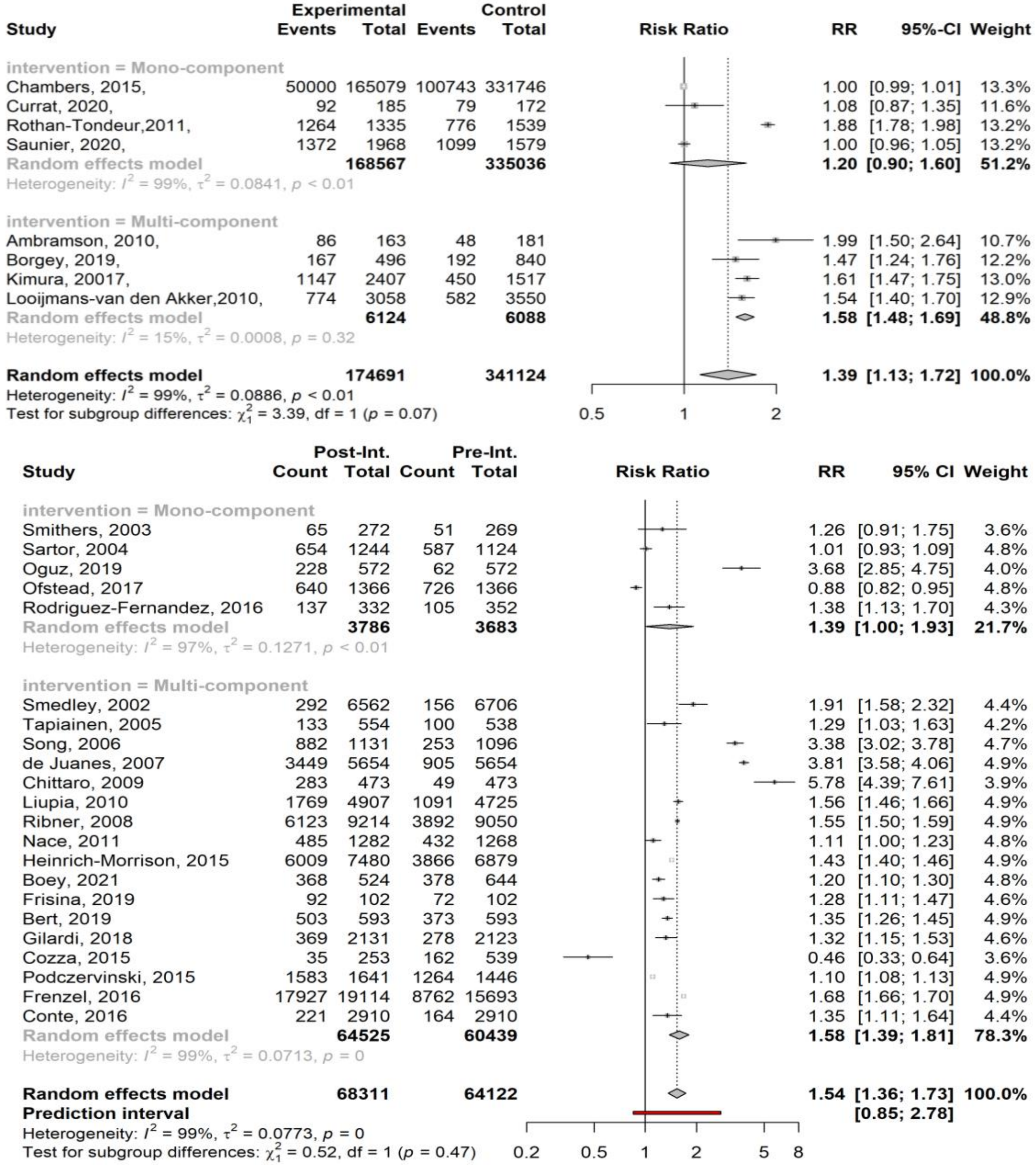

(b) Sensitivity analysis for Tdap vaccination (observational studies)

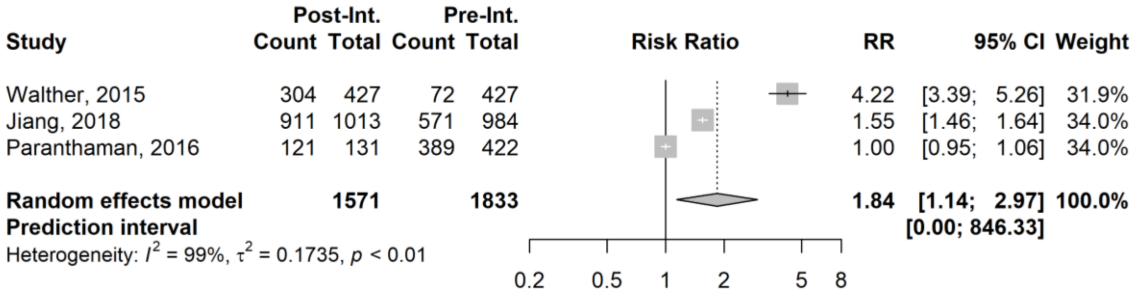

Supplement: Supplement [file 23-00276_COMORETTO_Supplement.pdf]
